# Synthesis of an Fe(terpy-cage)_2_ dumbbell†

**DOI:** 10.1039/d1ra08994c

**Published:** 2022-01-26

**Authors:** Frederic Dournel, Massoud Koshan, Philipp Woite, Michael Roemelt, Matthias Otte

**Affiliations:** Institut für Anorganische Chemie, Universität Göttingen Tammannstraße 4 37077 Göttingen Germany matthias.otte@chemie.uni-goettingen.de; Institut für Chemie, Humboldt-Universität zu Berlin Brook-Taylor Str. 2 12489 Berlin Germany michael.roemelt@hu-berlin.de

## Abstract

An azide masked amine is used to obtain a cage of lower symmetry that possess one terpy-group in an *exo*-position. This group can coordinate to iron(ii), yielding selectively an easy to purify Fe(terpy-cage)_2_ dumbbell. The dumbbell can also be obtained in a one pot reaction, which proceeded without isolation of the *exo*-functionalized cage.

## Abstract 2

A masked amine building block is used to synthesize an organic cage that is *exo*-functionalized with one terpy group. Two *exo*-functionalized cages can be combined *via* iron-terpy coordination resulting in a cage dumbbell.

## Graphical abstract

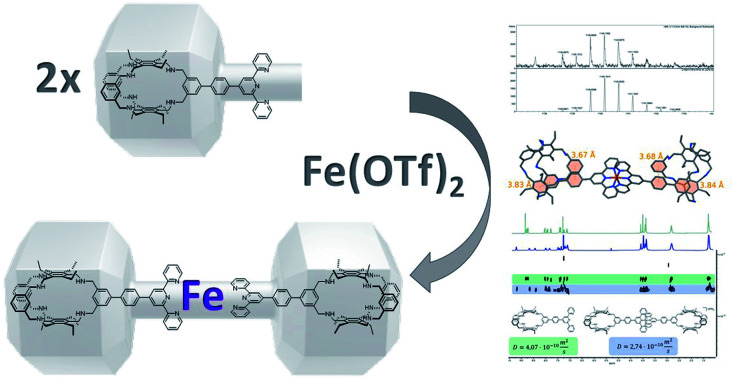

The synthesis of cavity-based species is of great interest in current chemistry. Examples range from macrocyclic- and cage-type compounds to frameworks.^1–3^ These species have been shown to be suitable for numerous applications such as molecular separation and catalysis.^4,5^

Combining organic cages and frameworks, Wang reported in 2019 on cage based covalent organic frameworks (COFs) that have been obtained *via* imine condensation reactions.^6^ These COFs possess a hexagonal skeleton with pillared cage nodes. Recently, Chen, Little and Cooper reported three-dimensional cage COFs that have also been obtained *via* imine condensation.^7^

Inspired by these findings, one may wonder about the possibility to selectively synthesize smaller fragments of cage-based frameworks. Instead of long range ordered frameworks, these compounds would be molecular species. The smallest possible unit would be the connection of two cages by a linker that results in a so-called cage dumbbell.

To obtain such structures, one could picture several synthetic approaches (Fig. 1). Mixing all building blocks in a single reaction step would represent the simplest synthetic pathway (Fig. 1a). The challenge is to overcome a statistical product distribution and to achieve high selectivity. Another approach towards cage dumbbells circumventing the statistical self-assembly problem is the connection of *exo*-functionalized cages *via* a linker (Fig. 1b). One may also think to make use of a macrocyclic precursor that selectively forms one cage, which can be linked to another (Fig. 1c).

**Fig. 1:**
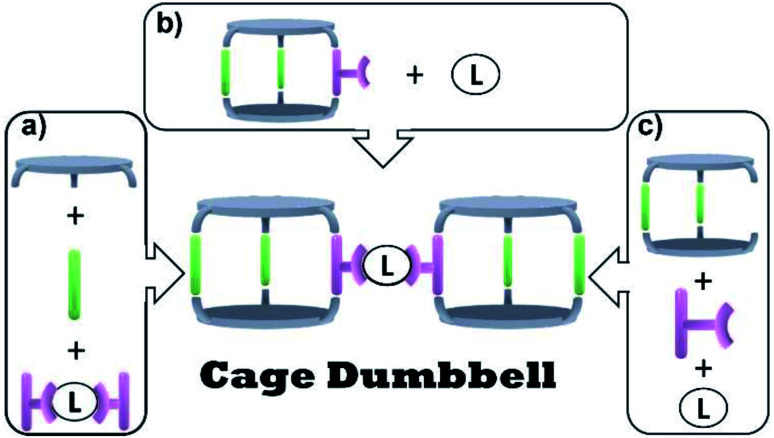
Conceptual synthetic approaches towards cage dumbbells. (a) One-pot approach; (b) connection of *exo*-functionalized cages *via* a linker; (c) *in situ* cage formation from a macrocycle and another building block that binds or contains the linker.

Greenaway recently followed the approach depicted in Fig. 1a. The synthesis of organic cage dumbbells *via* imine condensation was reported in that study. Thereby organic moieties were used as linker between two cages.^8^ Within the reported protocols, a tri-topic amine was reacted with a di-topic aldehyde and different tetra-topic aldehydes. The di-topic aldehyde was used in excess to steer the selectivity towards the desired dumbbell, as it competes with polymer formation. Unfortunately, the undesired organic cage, which is obtained *via* the reaction of three equivalents of di-topic aldehyde and two equivalents of tri-topic amine, was obtained as the major product. The desired dumbbell was only found in a small yield while being accompanied with insoluble poly- and oligomers. However, the cage-dumbbell could be purified *via* HPLC.

We reported previously protocols to synthesize organic cages of lower symmetry and *endo*-functionalized cages suitable to coordinate to transition-metals.^9,10^ In a more recent study, we combined both approaches and showed that an *endo*-functionalized cage of lower symmetry can perform heteroleptic ligation to iron and zinc.^11^ We were wondering if selective *exo*-functionalization of organic cages may be accomplished by using our developed synthetic strategy. We envision that this may lead to new possibilities such as the selective connection of two cages to form a cage dumbbell. This would follow the conceptual approaches depicted in Fig. 1b and c.

Terpyridine motifs are often applied for the synthesis of complex supramolecular species.^12^ As such, they have been used to construct macrocyclic and cage type compounds.^13,14^ Furthermore, they have been used to design molecular machines and metallo-helicoids.^15,16^ We were interested in a metal ion that forms diamagnetic complexes and can therefore be investigated *via* NMR. As such, we decided to peruse the synthesis of a Fe(terpy-cage)_2_ dumbbell *via* the combination of a reductive amination strategy and metal–ligand coordination. We chose macrocycle 1 and terpy-functionalized building block 2 as substrates for the Fe(terpy-cage)_2_ dumbbell synthesis (Scheme 1).

**Scheme 1:**
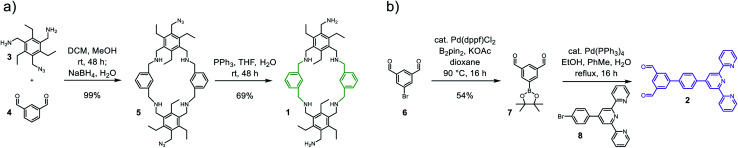
Synthesis of building blocks (a) 1 and (b) 2.

Starting from literature known 3,^17^ macrocycle 1 is obtained within two steps (Scheme 1a). The reductive amination of 3 and di-topic aldehyde 4 gives macrocycle 5 in 99% yield. A subsequent Staudinger reaction converts 4 into 1 with a yield of 69%. The azide acts here as a masked amine, preventing undesired cage formation during the reaction with 4. 1 was characterized *via* ESI-MS, ^1^H, ^13^C NMR and IR spectroscopy. The ESI-MS reveals signals at 1407.1 and 703.5426 corresponding to 1+H^+^ and 1+2H^+^. The ^1^H NMR shows no signal in the region of an aldehyde. Instead, three singlets that integrate to 8, 4 and 8 protons are found at 3.88, 3.82 and 3.72 ppm (Fig. 2a). These three signals correspond to the three benzylic CH_2_-groups in 1. 2 is obtained in two steps from commercially available 6 (Scheme 1b). A palladium-catalysed borylation of arylbromide 6 gives the Bpin-functionalized dialdehyde 7 (54%). A following Suzuki-coupling of 7 with 8 gives 2 (61%). The ^1^H NMR spectrum of 2 shows a singlet at 10.82 ppm corresponding to the aldehyde moieties and a signal set in the aromatic region that is well known for terpy-moieties (Fig. 2b).

**Fig. 2:**
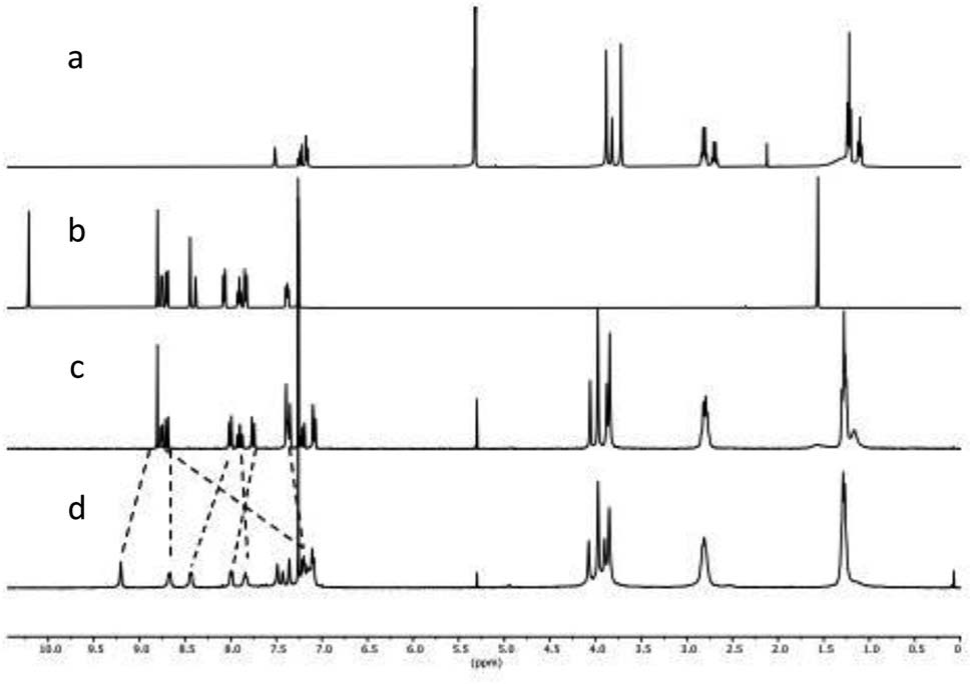
NMR spectra of (a) 1 in CD_2_Cl_2_, (b) 2 in CDCl_3_, (c) 9 in CDCl_3_ and (d) 10(OTf)_2_ in CDCl_3_.

With building blocks 1 and 2 in hand, we set out for the synthesis of the Fe(terpy-cage)_2_ dumbbell following the approaches shown in Fig. 1b and c (Scheme 2). We started with the stepwise dumbbell synthesis, which proceeds *via* the *exo*-functionalized cage 9. The reductive amination of 1 and 2 with NaBH_4_ gave the *exo*-functionalized cage 9 with 80% yield after purification *via* column chromatography. 9 was characterized *via* ESI-MS, ^1^H, ^1^H DOSY, ^13^C NMR and IR spectroscopy. The ^1^H NMR shows that the aldehyde signal has vanished and four singlets, corresponding to the four benzylic methylene groups, appear at 4.06, 3.97, 3.88 and 3.85 ppm (Fig. 2c). The aromatic region of the spectrum shows that the terpy motive is present in 9. Overall, the number and multiplicity of the signals observed matches with the expectations for 9. ^1^H DOSY NMR shows that, except for residual solvent signals, all observed signals belong to one species (see Fig. 3, green part and ESI). For this species, a diffusion coefficient of 4.07 × 10^−10^ m^2^ s^−1^ was obtained in CDCl_3_. Due to the terpy substituent and the arene connecting the terpy to the cage, the shape of 9 cannot be considered spherical, therefore a hydrodynamic radius is not reported. The ESI-MS reveals signals at 1112.6941, 557.3531 and 371.5710 corresponding to 9+H^+^, 9+2H^+^ and 9+3H^+^.

**Scheme 2:**
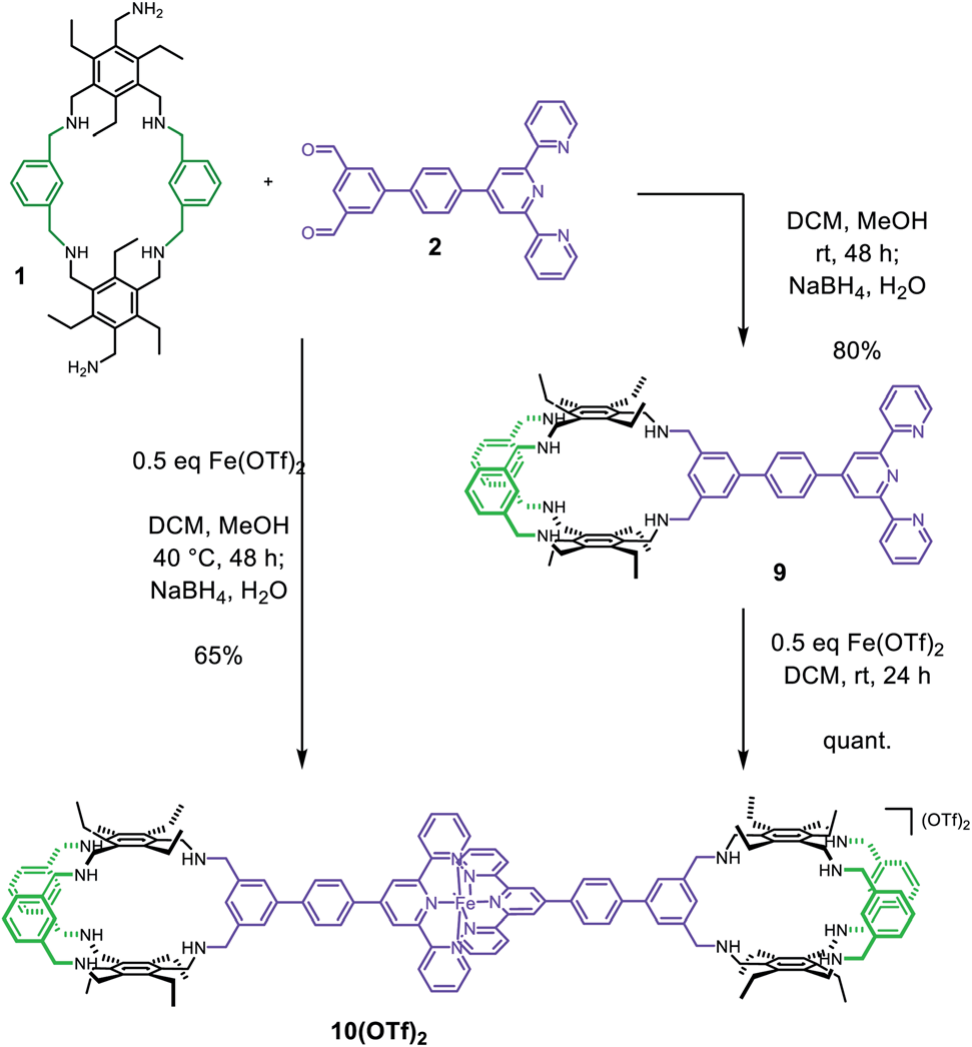
Synthesis of the Fe(terpy-cage)_2_ dumbbell 10^2+^*via*9 or directly from 1 and 2.

**Fig. 3:**
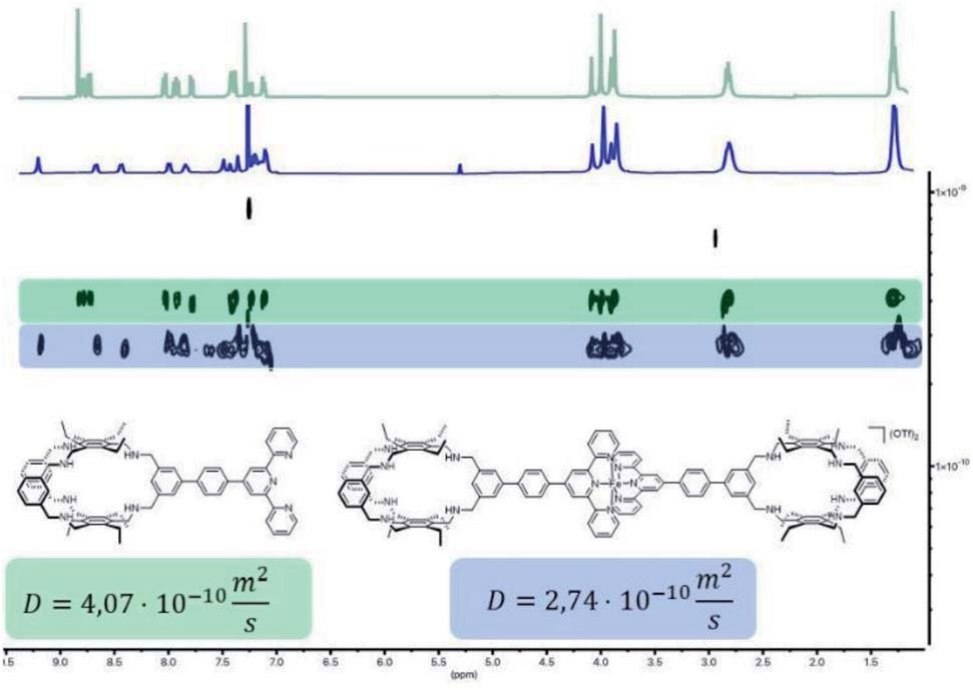
Superimposed ^1^H DOSY NMR spectra of 9 (green) and 10(OTf)_2_ (blue) in CDCl_3_.

After addition of half an equivalent of iron(ii) triflate to 9 in dichloromethane, the reaction mixture immediately turned purple, as one would expect for the formation of a Fe(terpy)_2_-complex. Removal of the solvent gave 10(OTf)_2_ in quantitative yield. The ESI-MS reveals signals at 1138.6675, 759.4385, 569.8295 and 456.0617 corresponding to 10^2+^, 10^2+^+H^+^, 10^2+^+2H^+^ and 10^2+^+3H^+^. The recorded UV/vis spectrum of 10(OTf)_2_ in dichloromethane shows a characteristic absorption band at *λ* = 574 nm, which by comparison with the parent [Fe(terpy)_2_]^2+^-complex could be assigned to a MLCT transition.^18^ The other two absorption bands at *λ* = 324 nm and 286 nm were assigned to π–π* transitions.^15^ Comparing the ^1^H NMR of 10(OTf)_2_ (Fig. 2d) and 9 (Fig. 2c) further reveals coordination of iron to the terpy motif. The signals belonging to the terpy moiety and the phenyl ring that connects the terpy with the cage shift due to complex formation (dotted lines between Fig. 2c and d). ^1^H DOSY NMR of 10(OTf)_2_ show that all signals belong to one species with a diffusion coefficient of 2.74 × 10^−10^ m^2^ s^−1^ (see Fig. 3, blue part and ESI†). Comparison of the diffusion coefficients of 9 with 10(OTf)_2_ indicates the expected increased size for 10(OTf)_2_. 9 and 10(OTf)_2_ are both soluble in chlorinated solvents such as dichloromethane and chloroform. Interestingly, 9 is not soluble in MeOH, while 10(OTf)_2_ is very well (see the ESI† for a ^1^H NMR of 10(OTf)_2_ in CD_3_OD).

The structural properties of 10^2+^ were investigated by means of quantum chemical calculations. As expected, the strong metal–ligand bonds ensure an almost perfectly linear connection between the two cage units throughout all of our simulations. Furthermore, our results indicate that in both cages the functionalized arm rotates around its amine bonds such that it allows a parallel alignment of its aryl ring and one of the aryl rings at the top or bottom of the cage as depicted in Fig. 4. In a similar fashion the rotational flexibility of the amine bonds of another arm enables the parallel alignment of the aryl ring that connects the cage to the Fe(terpy)_2_ unit with the aryl of ring this arm. Therefore, the proposed structures are stabilized by 4 π–π interactions. Alternative arrangements of 10^2+^ feature one or multiple solvent molecules inside the cage units thereby establishing a less skewed arrangement of the cage units and impeding the formation of stacked π systems at the same time (see ESI†). However, in our simulations such structures were energetically unfavorable owing to the lack of considerable direct interactions between the solvent and 10^2+^. Of course, one should keep in mind that the relative energies of the different structural isomers depend on the nature of the solvent and the experimental conditions. According to our optimizations, the structure of the cage elements in 10^2+^ largely resemble the structure of the isolated cages 9. Most importantly, the aforementioned π–π interaction is retained upon formation of 10^2+^. An overlay of the structure obtained from fusing optimized structures of 9 and [Fe(terpy-Ph)_2_]^2+^ (see ESI for details†) and the optimized structure of 10^2+^ shown in Fig. S31† visualizes the close structural resemblance within the cage units. 10^2+^ is a fairly flexible compound which is highlighted by 230 conformers predicted to be within an energy range of 5 kcal mol^−1^. Fig. S29† depicts an overlay of these 230 structures.

**Fig. 4:**
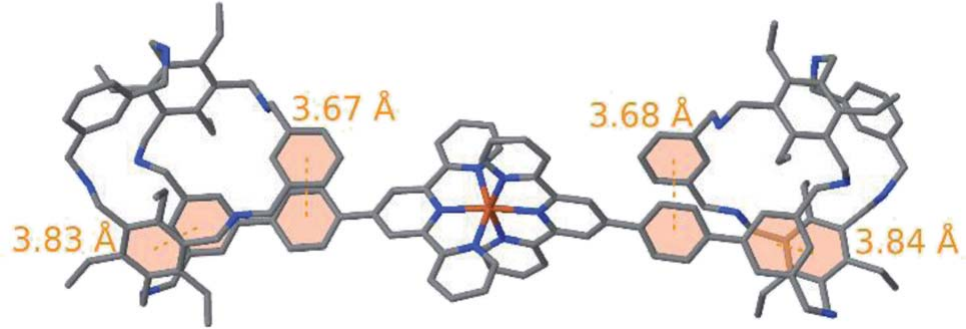
Optimized structure of 10^2+^. Rotation of two arms around their amine bonds allows for the parallel alignment of two pairs of aryl rings leading to π–π interactions. The involved aryl rings are highlighted in orange and connected by dotted lines.

Finally, we set out for the direct synthesis of 10(OTf)_2_ from 1 and 2. To our delight, the reductive amination protocol does not (or only barely) interfere with the iron-coordination. As such 10(OTf)_2_ could be isolated with a 65% yield, compared to the 80% that have been obtained *via* the two step approach. This shows that our synthetic strategy to low symmetry cages combined with metal-terpy coordination selectively leads to cage dumbbells.

In summary, we report here the synthesis of the Fe(terpy-cage)_2_ dumbbell 10(OTf)_2_. 10(OTf)_2_ is selectively obtained *via* a stepwise approach over *exo*-functionalized cage 9. In addition, we demonstrated that 10(OTf)_2_ can also be obtained *via* a direct approach from building blocks 1 and 2. Thereby, we used our already established synthetic protocol for cages of lower symmetry that uses building block 3, which introduces an azide masked amine. We combined this protocol with the well-known iron-terpy coordination chemistry. We believe that this approach may be used to synthesize a variety of different cage-connected and defined subunits of polymeric framework structures.

## Conflicts of interest

There are no conflicts to declare.

## Supplementary Material

RA-012-D1RA08994C-s001
