# Supplementary material for: Synthesis of an Fe(terpy-cage)2 dumbbell
Source: RSC Adv. 2022 Jan 26;12(6):3402–5. doi: 10.1039/d1ra08994c (PMC8979284; doi:10.1039/d1ra08994c)
Supplement: RA-012-D1RA08994C-s001 [file RA-012-D1RA08994C-s001.pdf]

Electronic Supplementary Material (ESI) for RSC Advances.
This journal is © The Royal Society of Chemistry 2022

Synthesis of an Fe(terpy-cage)$_2$ dumbbell

Frederic Dournel, ‡ [a] Massoud Koshan, ‡ [a] Philipp Woite,[b] Michael Roemelt[b]* and Matthias Otte[a]*

[a] Institut für Anorganische Chemie; Universität Göttingen; Tammannstraße 4, 37077 Göttingen. E-mail: matthias.otte@chemie.uni-goettingen.de
[b] Institut für Chemie, Humboldt-Universität zu Berlin, Brook-Taylor Str. 2, 12489 Berlin, Germany. E-mail: michael.roemelt@hu-berlin.de
† Electronic Supplementary Information (ESI) available: [details of any supplementary information available should be included here]. See DOI: 10.1039/x0xx00000x
‡ These authors contributed equally

Table of Contents

1. General Information **1**
2. Experimental Procedures **2**
2.1. Synthesis of **1** 2
2.2. Synthesis of **2** 2
2.3. Synthesis of **5** 3
2.4. Synthesis of **9** 3
2.5. Synthesis of **10(OTf)$_2$** 4
3. Spectra **5**
4. Computational Details **19**
5. References **21**

1. General Information

All reactions involving air- or moisture sensitive compounds were carried out under argon using either standard Schlenk and vacuum line techniques or in a UNILAB Glovebox from MBraun. All reagents were purchased from commercial sources and used as received unless otherwise stated. Methanol (MeOH) was degassed by sparging with argon and stored over molecular sieves under argon. Tetrahydrofuran (THF), dichloromethane (DCM) and Benzene were taken from an MBRAUN MB SPS-800 solvent purification system. Deuterated DCM was degassed using the freeze -pump-thaw cycles according to the procedure found in literature[1] and subsequently stored over molecular sieves.

^{1}H and ^{13}C NMR spectra were recorded on a Bruker Avance 300, Avance 400 or Avance 500 spectrometer at 25 °C. ^{1}H and ^{13}C NMR chemical shifts are reported in ppm relative to TMS using the residual solvent resonance as internal standard. Infrared spectra were recorded using an ALPHA Platinum-ATR FTIR spectrometer from Bruker. MS measurements were performed at the analytic laboratory of the chemistry department.

2. Experimental Procedures

Compounds **3**[1] and **7**[2] were synthesized as described in literature.

2.1 Synthesis of 1

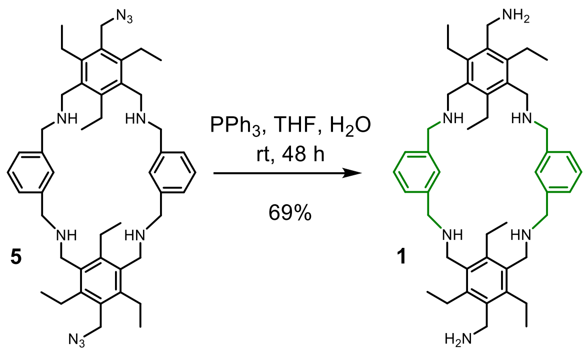

In a 100 mL round bottom flask, **5** (671 mg, 889 µmol, 1.00 eq) and triphenylphosphine (940 mg, 3.55 mmol, 4.00 eq) were dissolved in THF (20 mL) and stirred for 30 min at room temperature. After addition of Water (0.6 mL), the solution was stirred for 2 d at room temperature. The solvent was removed under reduced pressure and the crude product was purified via column chromatography over silica (gradient of DCM:MeOH = 70:30 to DCM:MeOH:7 N NH_3 in MeOH = 62:30:8) to afford **1** as a colourless solid (430 mg, 69%). 1H NMR (400 MHz, CD2Cl2): 7.51 (s, 2H), 7.24 (dd, J = 8.4 Hz, 6.4 Hz, 2H), 7.17 (dd, J = 6.7 Hz, 1.7 Hz, 4H), 3.88 (s, 8H), 3.82 (s, 4H), 3.72 (s, 8H), 2.81 (q, J = 7.5 Hz, 8H), 2.70 (q, J = 7.4 Hz, 4H), 1.22 (t, J = 7.5 Hz, 12H), 1.10 (t, J = 7.4 Hz, 6H). 13C NMR (101 MHz, CD_2Cl_2): 142.38, 141.83, 141.21, 137.45, 134.76, 128.22, 127.33, 127.22, 55.40, 47.92, 40.01, 22.93, 22.76, 17.15, 17.04. IR (neat): 2959, 2924, 2866, 2743, 1610, 1591, 1572, 1496, 1432, 1371, 1320, 1265, 1193, 1159, 1112, 1097, 1065, 1045, 996, 958, 944, 827, 778, 761, 736, 700, 675, 615, 584. Exact mass ESI MS: $C_{46}H_{66}N_6H$ calculated: 703.5422, found: 703,5426.

2.2 Synthesis of 2

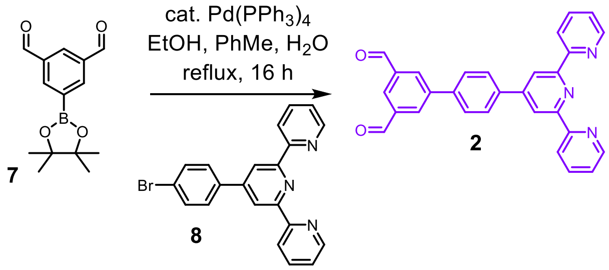

61%

In an oven-dried three-necked 100 mL round bottom flask equipped with a reflux condenser, **8** (1.62 g, 4.16 mmol 1.00 eq), **7** (1.19 g, 4.58 mmol, 1.10 eq) and sodium carbonate (17.9 g, 62.4 mmol, 15.00 eq) were dissolved in Toluene (40 mL), ethanol (20 mL) and water (20 mL) and degassed for 30 min. After addition of $Pd(PPh_3)_4$ (480 mg, 416 mmol, 0.10 eq), the reaction mixture was stirred for reflux for 18 h. The precipitate was then separated via filtration, washed with water (50 mL), ethyl acetate (50 mL) and toluene (50 mL) to afford **2** as a light grey solid (1.12 g, 61%). ^1H NMR (400 MHz, $CDCl_3$): 10.21 (s, 2H, H-1), 8.80 (s, 2H), 8.76 (d, *J* = 3.7 Hz), 8.70 (d, *J* = 7.9 Hz, 2H); 8.44 (s, 2H), 8.39 (s, 1H), 8.07 (d, *J* = 8.4 Hz, 2H); 7.90 (t, *J* = 7.2 Hz, 2H), 7.84 (d, *J* = 8.2 Hz, 2H), 7.42 – 7.34 (m, 2H). ^{13}C NMR (101 MHz, $CDCl_3$): 207.07, 191.13, 156.25, 149.31, 142.71, 139.05, 137.79, 137.08, 133.01, 130.06, 128.34, 127.81, 124.10, 121.55, 118.86. IR (neat): 3051, 3011, 2811, 2733, 1707, 1695, 1595, 1581, 1564, 1541, 1521, 1466, 1437, 1409, 1389, 1378, 1311, 1289, 1267, 1206, 1193, 1148, 1121, 1093, 1084, 1071, 1037, 1014, 1003, 988, 904, 894, 884, 848, 838, 818, 793, 745, 735, 718, 697, 688, 674, 659, 642, 622, 614, 584, 571, 545, 513. Exact Mass ESI MS: $C_{29}H_{20}N_3O_2H$ calculated: 442.1550 found: 442.1537.

2.3 Synthesis of 5

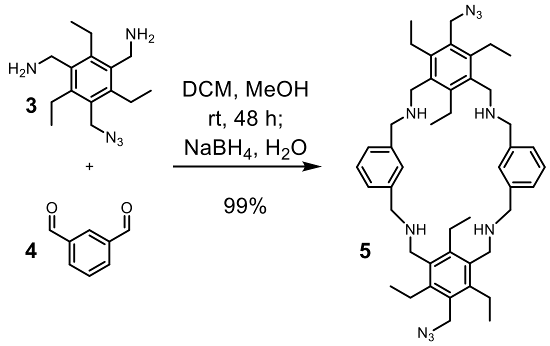

In an oven-dried Schlenk tube under argon atmosphere, **3** (600 mg, 2.18 mmol, 1.00 eq) and **4** (292 mg, 2.18 mmol, 1.00 eq) were dissolved in dichloromethane (30 mL) and methanol (10 mL) and stirred for 2 d at room temperature. After addition of sodium borohydride (412 mg, 10.9 mmol, 5.00 eq) the solution was stirred for additional 3 h at room temperature. The solution was then extracted with dichloromethane (3x10 mL) and water (3x10 mL). The combined organic phases were dried over anhydrous magnesium sulfate and the solvent was removed under reduced pressure. The product was isolated as a colorless solid (819 mg, 99%). ^{1}H NMR (400 MHz, CD_2Cl_2): 7.52 (s, *J* = 1.9 Hz, 2H), 7.25 (dd, *J* = 8.4 Hz, 6.5 Hz, 2H), 7.20 – 7.14 (m, 4H), 4.46 (s, 4H), 3.89 (s, 8H), 3.73 (s, 8H), 2.81 (q, *J* = 7.5 Hz, 8H), 2.72 (q, *J* = 7.4 Hz, 4H), 1.21 (t, *J* = 7.5 Hz, 12H), 1.09 (t, *J* = 7.5 Hz, 6H). ^{13}C NMR (101 MHz, CD_2Cl_2): 144.52, 143.11, 141.14, 135.05, 129.40, 128.30, 127.38, 127.32, 55.29, 48.80, 47.78, 23.19, 22.88, 16.88, 16.66. IR (neat): 3319, 2962, 2927, 2868, 2085, 1608, 156. Exact mass ESI MS: $C_{46}H_{62}N_{10}H$ calculated: 755,5232 found: 755,5232.

2.4 Synthesis of 9

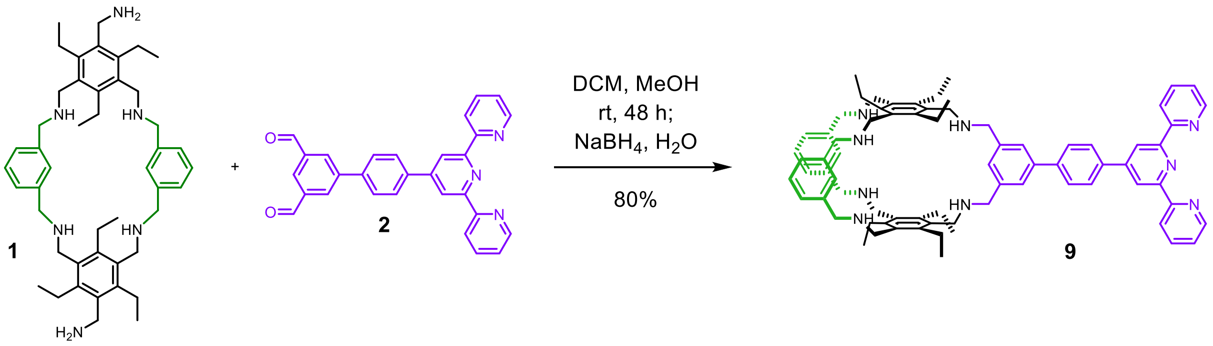

In an oven-dried Schlenk tube under argon atmosphere, **1** (251 mg, 357 µmol, 1.00 eq) and **2** (158 mg, 357 µmol, 1.00 eq) were dissolved in dichloromethane (15 mL) and methanol (15 mL) and stirred for 3 d at room temperature. Sodium borohydride (13.5 mg, 357 µmol, 1.00 eq) was added and the solution was stirred for additional 2 h at room temperature. The solution was extracted with water (3x10 mL) and dichloromethane (3x10 mL) and dried over anhydrous magnesium sulfate. After removal of the solvent under reduced pressure, the crude product was purified via column chromatography over silica (DCM:MeOH:7N NH_3 in MeOH = 95:3:2) to afford **9** as a colorless solid (317 mg, 80%). ^{1}H NMR (300 MHz, $CDCl_3$): 8.80 (s, 2H), 8.76 (ddd, *J* = 4.8 Hz, 1.8 Hz, 0.9 Hz, 2H), 8.70 (dd, *J* = 8.0, 1.1 Hz, 2H), 8.00 (d, *J* = 8.4 Hz, 2H), 7.90 (td, *J* = 7.7, 1.8 Hz, 2H), 7.75 (d, *J* = 8.3 Hz, 2H), 7.42 – 7.32 (m, 5H), 7.37 (dd, *J* = 12.3 Hz, 1.2 Hz. 2H), 7.22 (dd, *J* = 8.3 Hz, 6.8 Hz, 2H), 7.13 – 7.04 (m, 4H), 4.06 (s, 4H), 3.97 (s, 8H), 3.88 (s, 4H), 3.85 (s, 8H), 2.85 – 2.75 (m, 12H), 1.33 – 1.21 (m, 18H). ^{1}H DOSY NMR: $D = 4.07 \cdot 10^{-10}$ $m^2 \cdot s^{-1}$. ^{13}C NMR: (101 MHz. $CDCl_3$): 156.15, 155.82, 149.00, 142.25, 140.75, 139.97, 136.73, 134.05, 127.49, 125.60, 124.58, 123.68, 121.24, 118.54, 55.02, 54.88, 48.24, 22.60, 16.53. IR (neat): 3307, 2962, 2927, 2867, 1732, 1641, 1602, 1583, 1566, 1517, 1465, 1442, 1407, 1388, 1373, 1319, 1319, 1263, 1189, 1114, 1067, 990, 906, 833, 793, 770, 739, 698, 660, 622, 586. Exact mass ESI-MS: $C_{75}H_{85}N_9$ calculated: 1112,7001, found: 1112,6941.

2.5 Synthesis of 10(OTf)$_2$

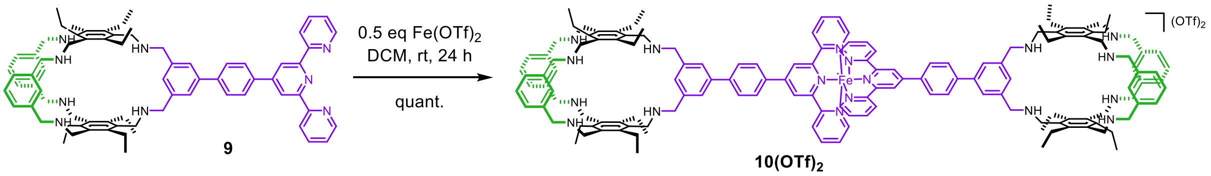

In a glovebox, **9** (67.1 mg, 60.3 µmol, 2.00 eq) was placed in a vial and dissolved in dichloromethane (2 mL). After addition of Iron(II)-trifluoromethanesulfonate (10.7 mg, 30.1 µmol, 1.00 eq), the reaction mixture was stirred for 1 d at room temperature. The solvent was removed under reduced pressure to afford **10(OTf)$_2$** as a purple solid (77 mg, quant.). ^{1}H NMR (600 MHz, MeOD): 9.52 (s, 4H), 8.88 (dt, *J* = 8.0 Hz, 1.0 Hz, 4H), 8.54 (d, *J* = 8.5 Hz, 4H), 8.12 (d, *J* = 8.1 Hz, 4H), 8.00 (td, *J* = 7.7 Hz, 1.5 Hz, 4H), 7.70 (d, *J* = 1.6 Hz, 4H), 7.35 (dt, *J* = 5.7 Hz, 0.9 Hz, 4H), 7.33 (t, *J* = 1.5 Hz, 2H), 7.30 – 7.23 (m, 8H), 7.24 – 7.19 (m, 12H), 3.99 (s, 8H), 3.92 (s, 8H), 3.89 – 3.84 (m, 32H), 2.88 – 2.69 (m, 24H), 1.20 (m, 36H). ^{1}H DOSY NMR: $D = 2.74 \cdot 10^{-10}$ m$^2 \cdot$ s^{-1}. ^{13}C NMR (151 MHz, $CDCl_3$): 161.88, 159.71, 154.03, 151.91, 144.70, 143.88, 142.38, 141.25, 141.11, 140.12, 136.77, 134.49, 134.40, 129.55, 129.32, 129.30, 128.78, 127.99, 127.56, 127.44, 126.71, 125.29, 122.86, 122.47, 120.75, 55.41, 55.34, 30.67, 23.85, 17.21. IR (neat): 2864, 1595, 1442, 1256, 1152, 1029, 830, 788, 637, 571, 517, 501. UV/vis (in DCM): 574, 324, 286. Exact mass ESI MS: $C_{150}H_{170}FeN_{18}$ calculated: 1139,6598, found: 1139,6609).

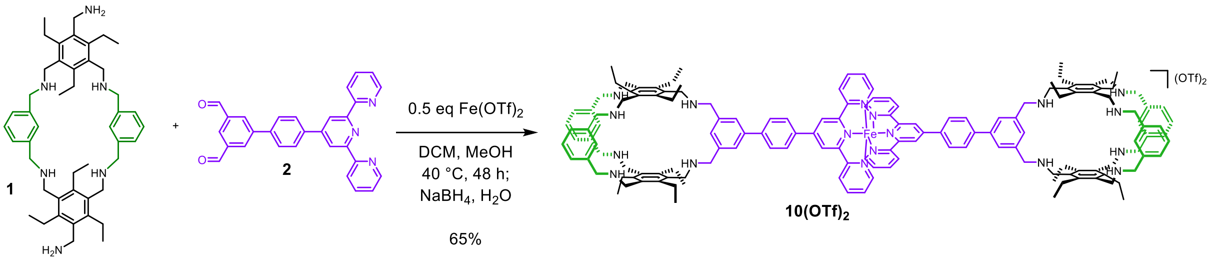

An oven-dried Schlenk tube under argon atmosphere was charged with **1** (60.0 mg, 85.3 µmol, 2.00 eq), **2** (37.6 mg, 85.3 µmol, 2.00 eq) and iron(II)-trifluoromethanesulfonate (15.1 mg, 42.7 µmol, 1.00 eq). Dichloromethane (24 mL) and methanol (6 mL) were added and the resulting solution was stirred for 2 d at 40 °C. After addition of sodium borohydride (12.9 mg, 341 µmol, 4.00 eq) and stirring for additional 3 h at room temperature, the solution was extracted with water (3x20 mL) and dichloromethane (3x30 mL) and dried over anhydrous magnesium sulfate. The crude product was dissolved in dichloromethane (5 mL) and *n*-Pentane (50 mL) were added and the precipitate was filtrated. This procedure was repeated 5 times to afford pure **10(OTf)$_2$** as a purple solid (65%, average value from 3 experiments).

3 Spectra

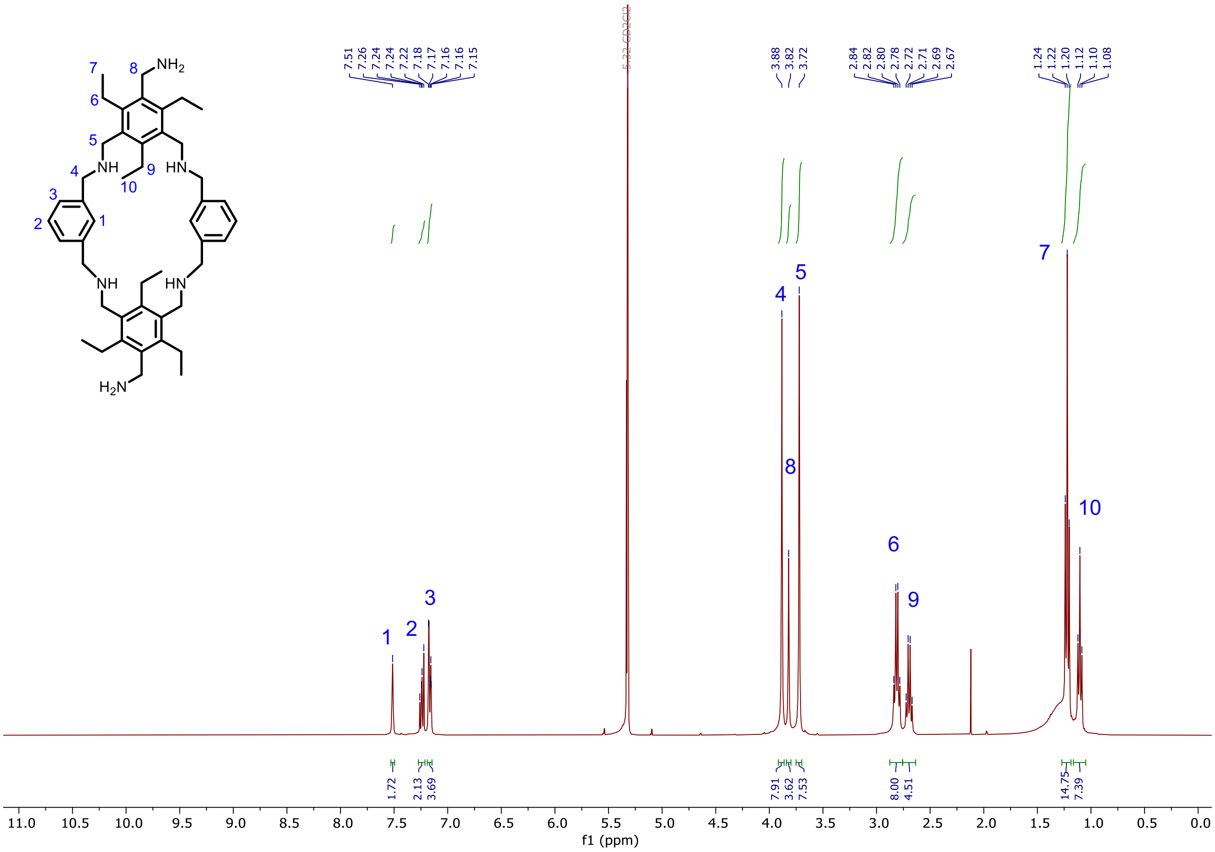

Figure S1. ^{1}H NMR spectrum of **1** in CD_2Cl_2 at rt.

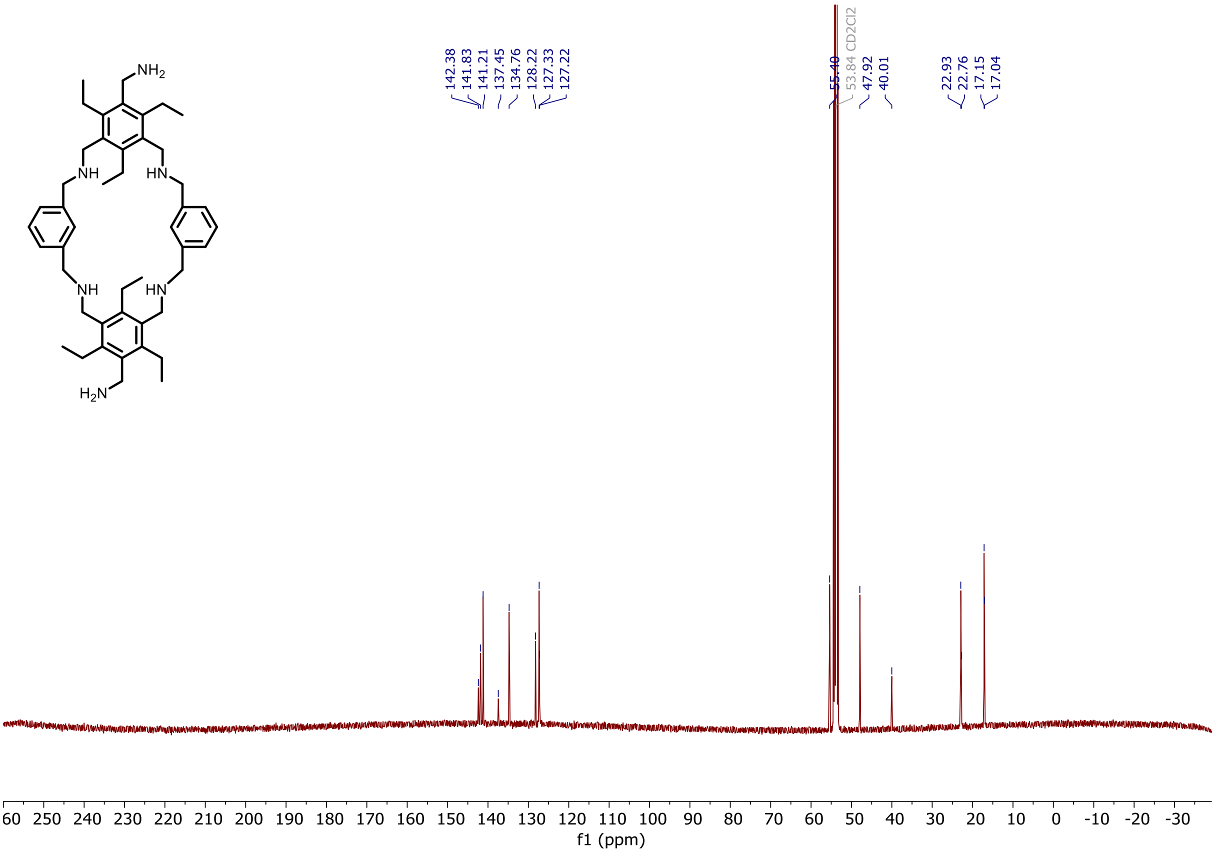

Figure S2. ^{13}C NMR spectrum of **1** in CD_2Cl_2 at rt.

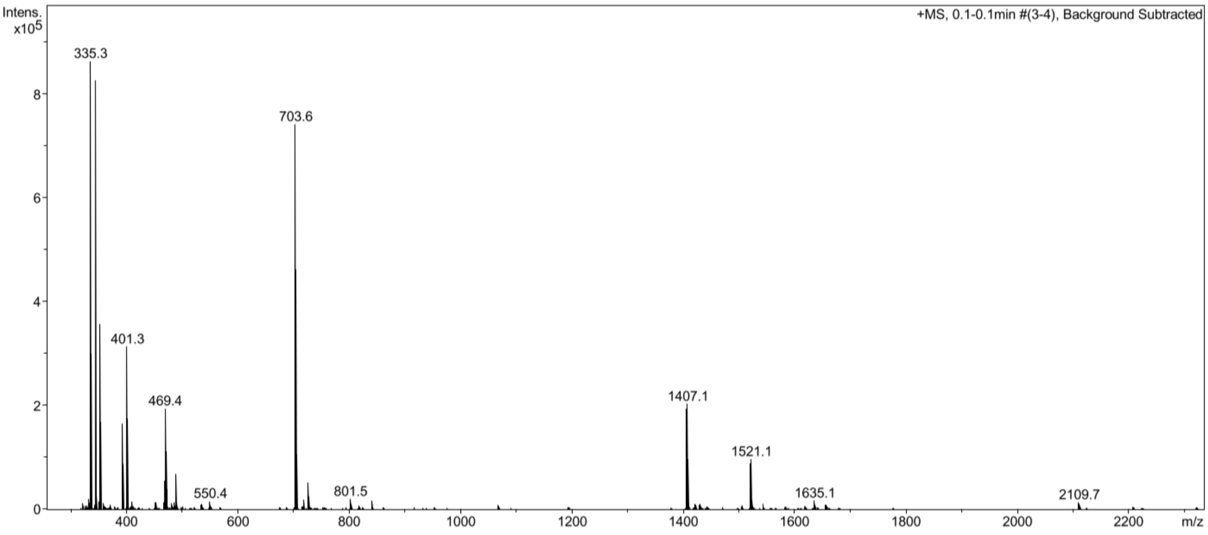

Figure S3. ESI MS spectrum of **1**.

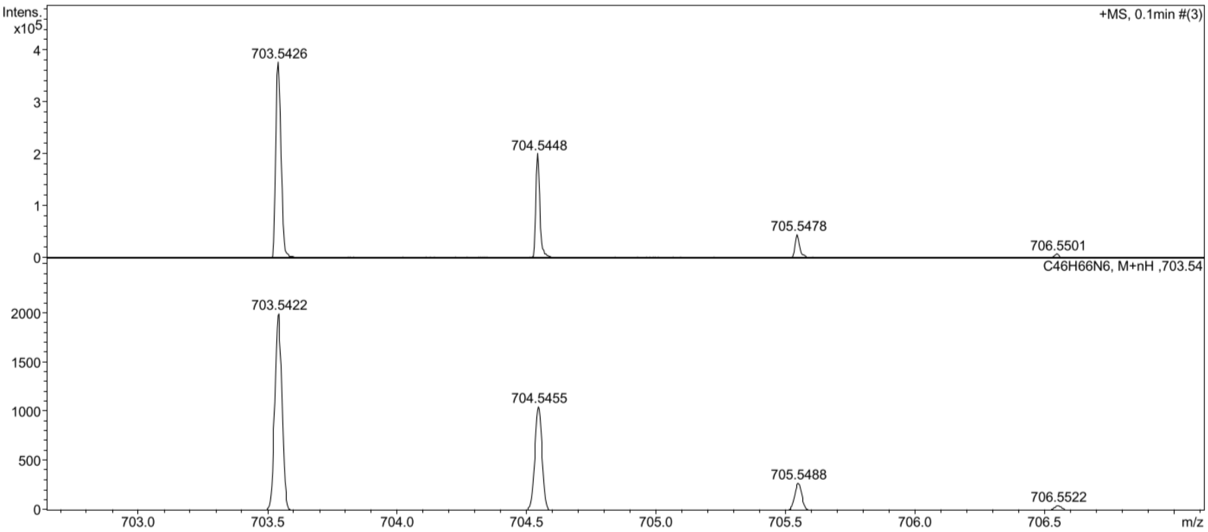

Figure S4. Measured and calculated ESI MS of **1** for $[M+H]^+$.

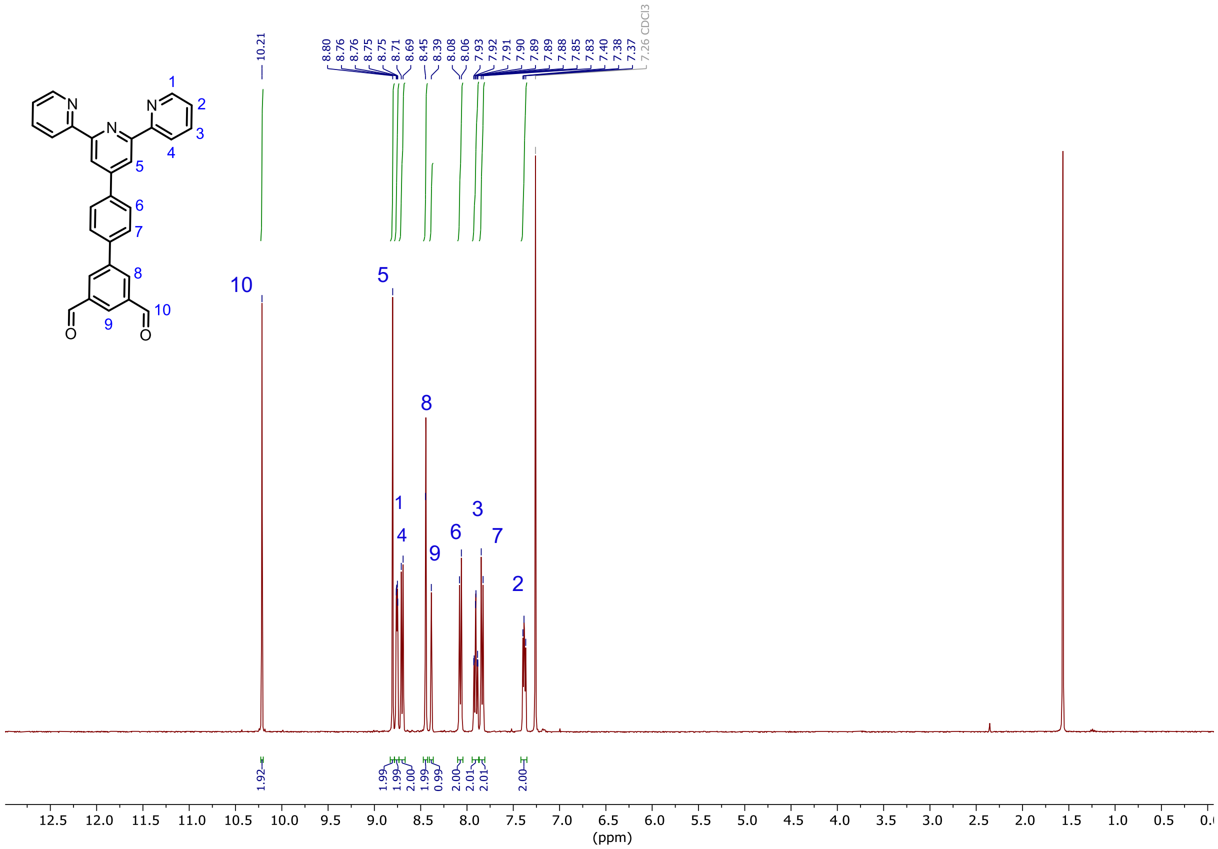

Figure S5. ^{11}H NMR spectrum of **2** in CDCl3 at rt.

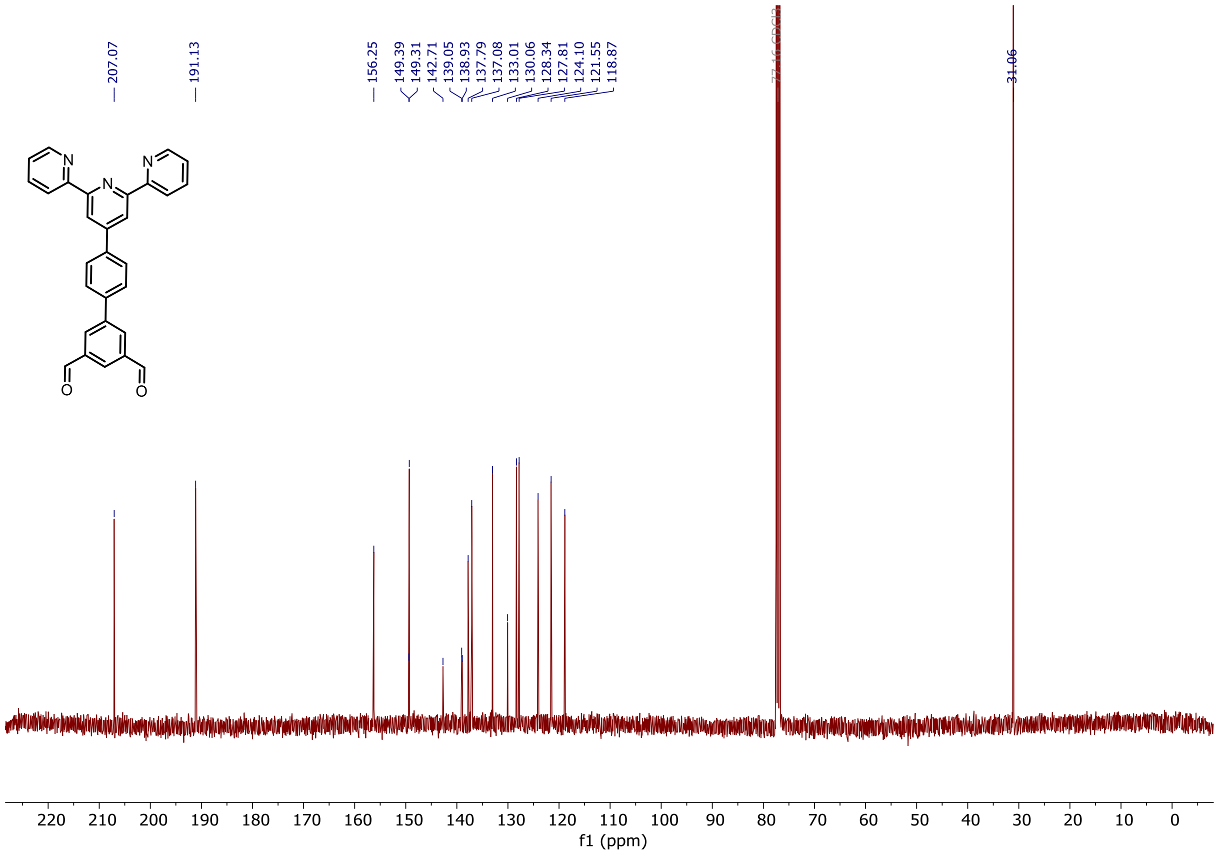

Figure S6. ^{13}C NMR spectrum of **2** in $CDCl_3$ at rt.

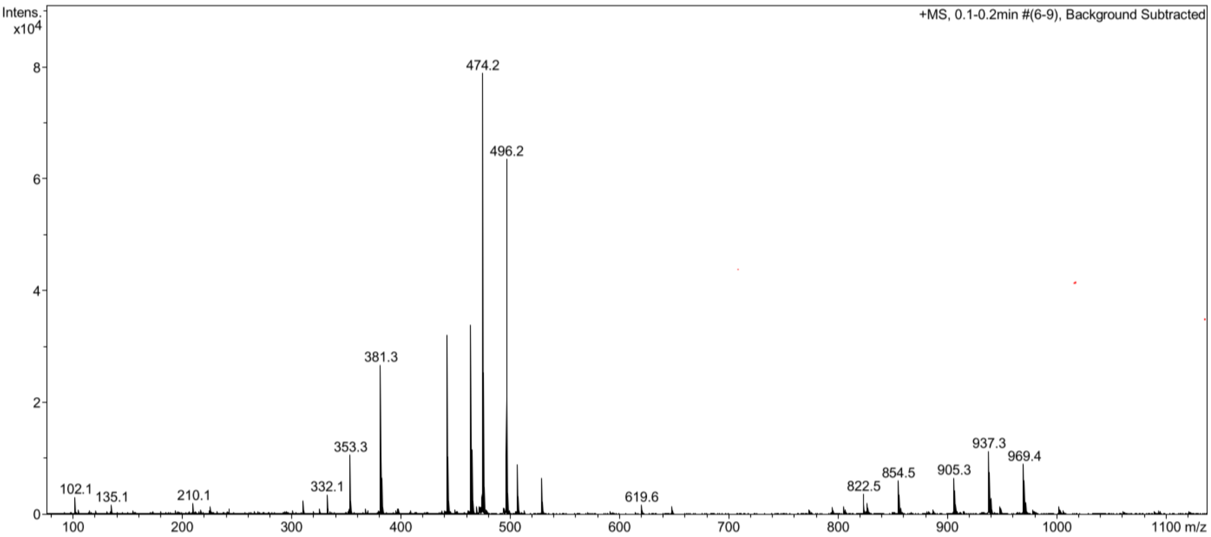

Figure S7. ESI MS spectrum of **2**.

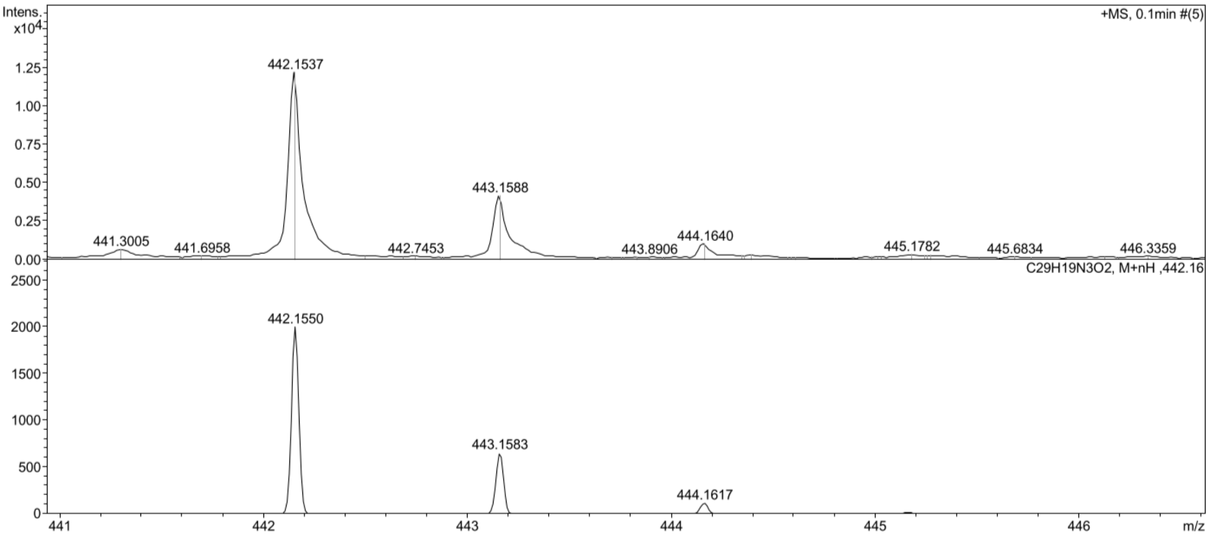

Figure S8. Measured and calculated ESI MS of **2** for $[M+H]^+$.

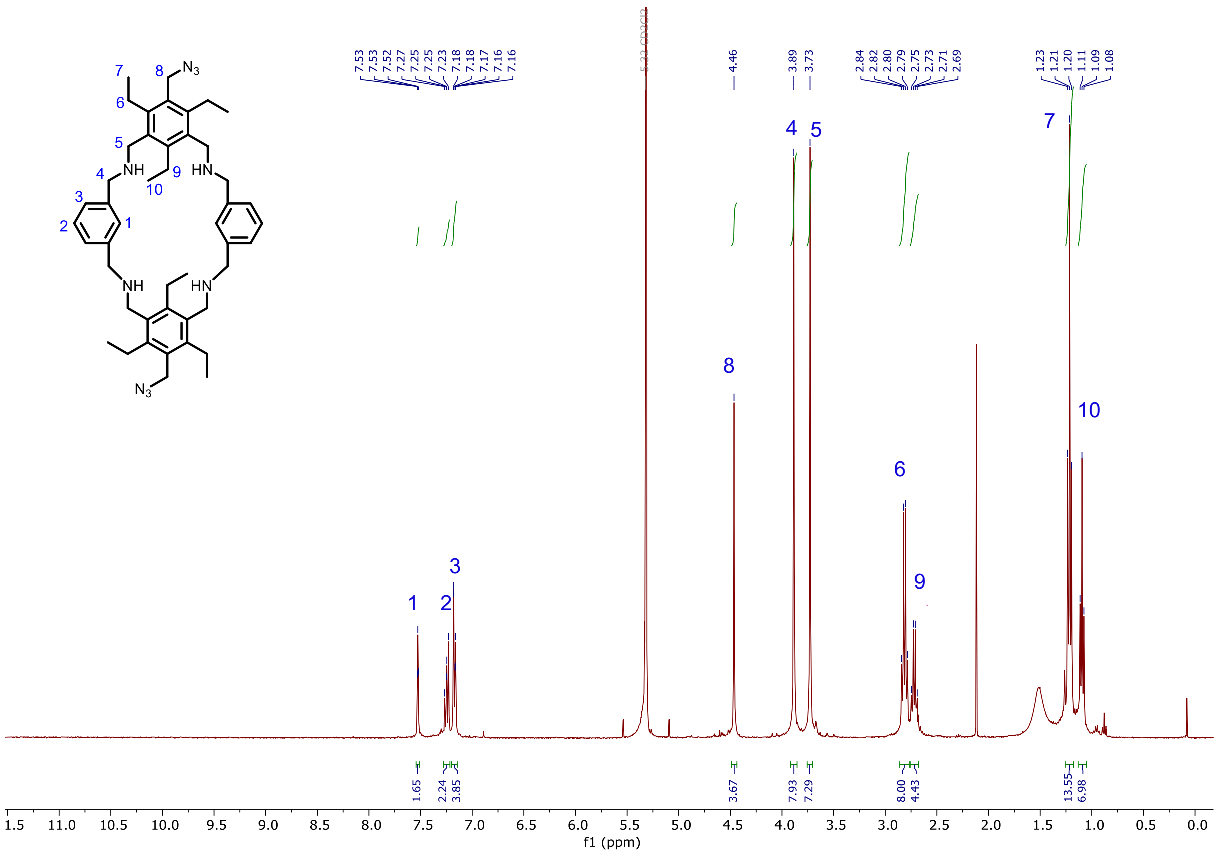

Figure S9. ^{1}H NMR spectrum of **5** in CD_2Cl_2 at rt.

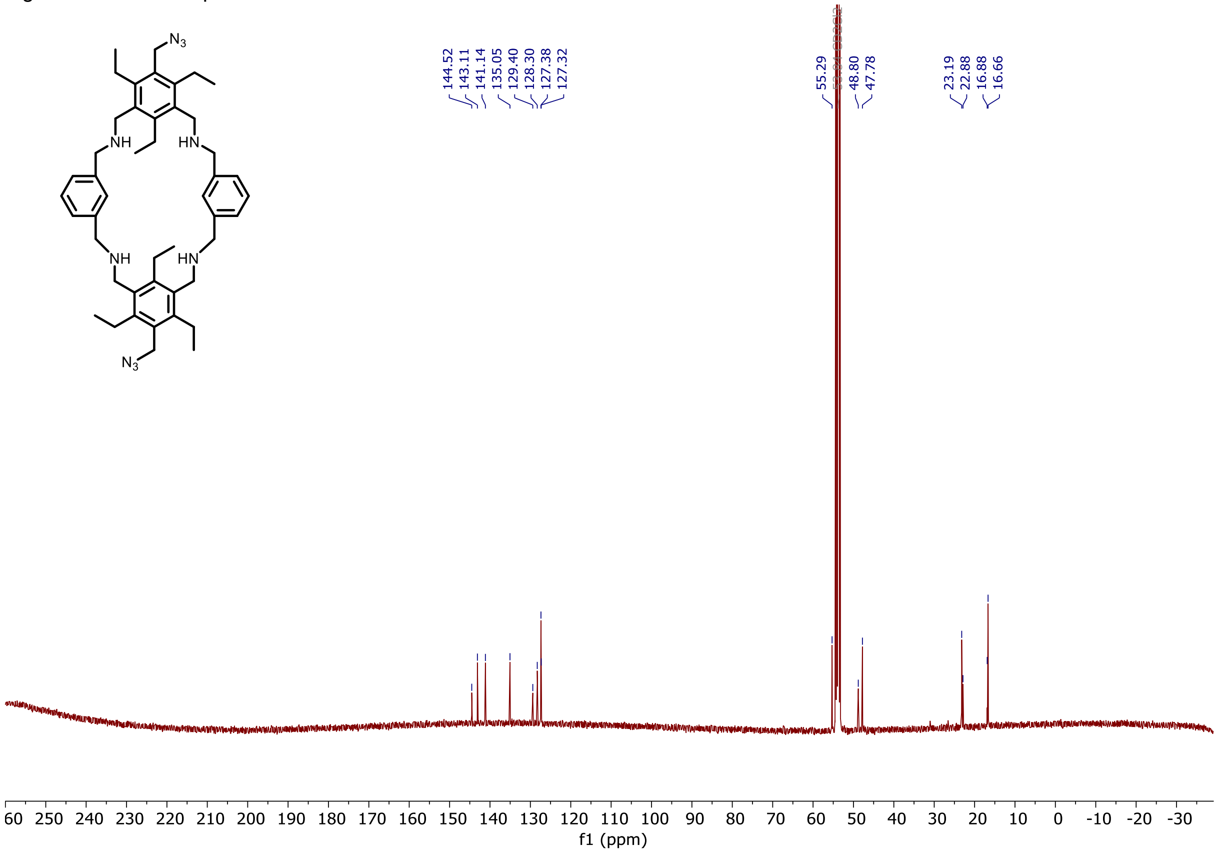

Figure S10. ^{13}C NMR spectrum of **5** in CD_2Cl_2 at rt.

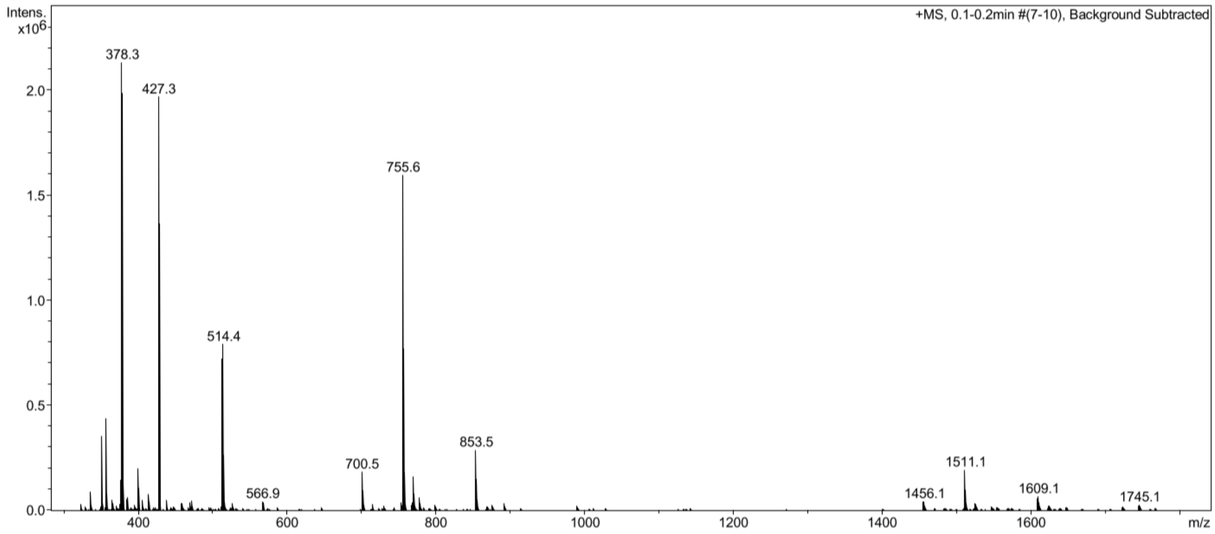

Figure S11**.** ESI MS spectrum of **5**.

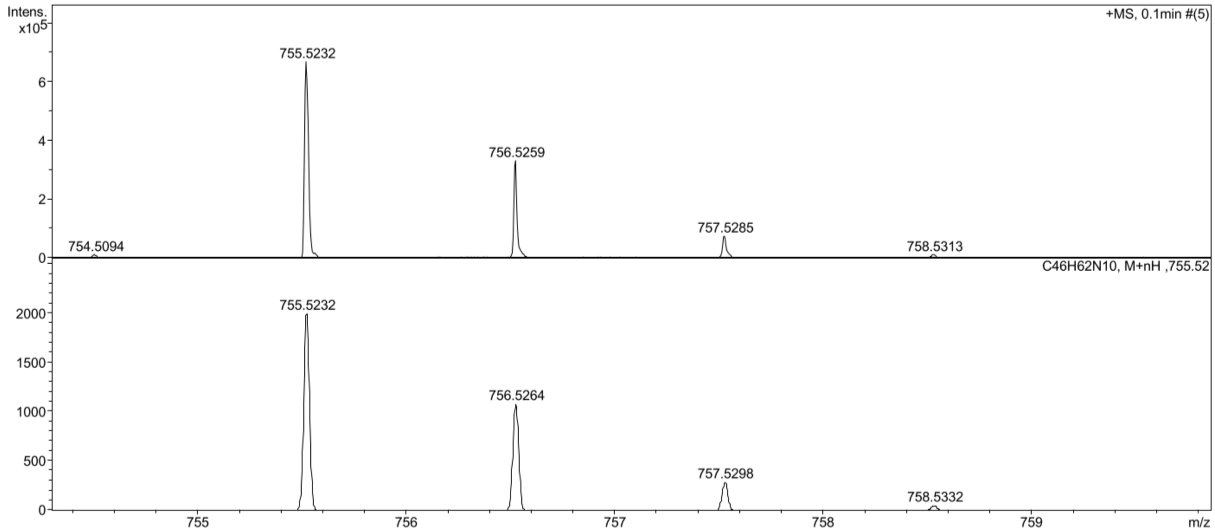

Figure S12**.** Measured and calculated ESI MS of **5** for $[M+H]^+$.

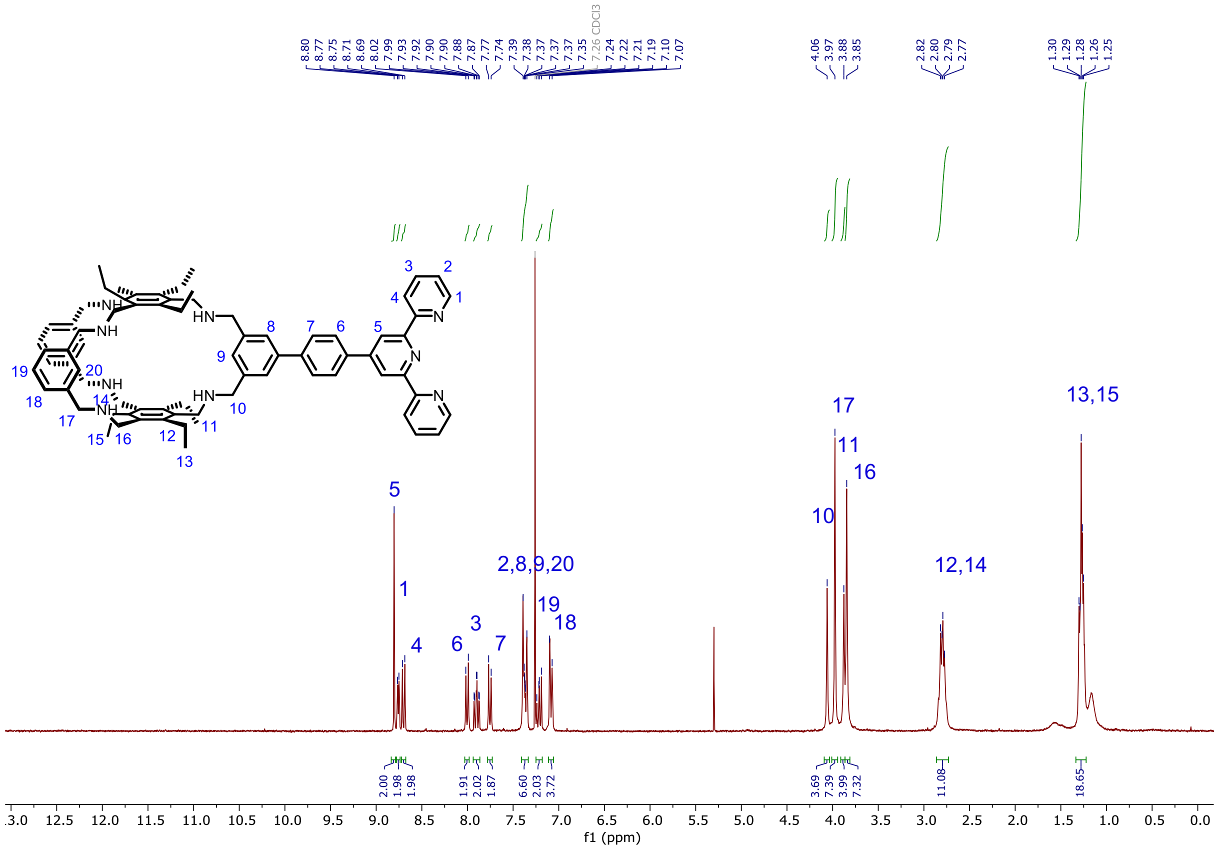

Figure S13. ^{1}H NMR spectrum of **9** in $CDCl_3$ at rt.

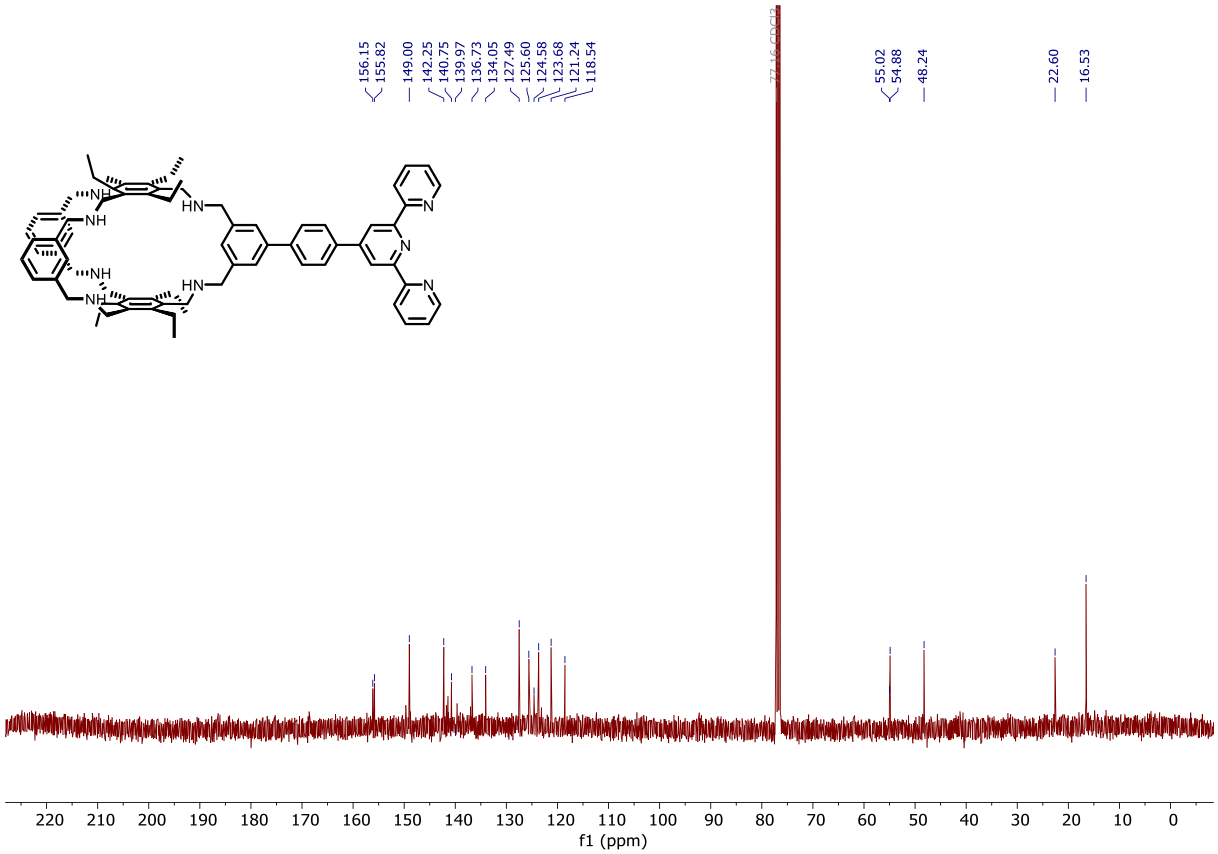

Figure S14. ^{13}C NMR spectrum of **9** in $CDCl_3$ at rt.

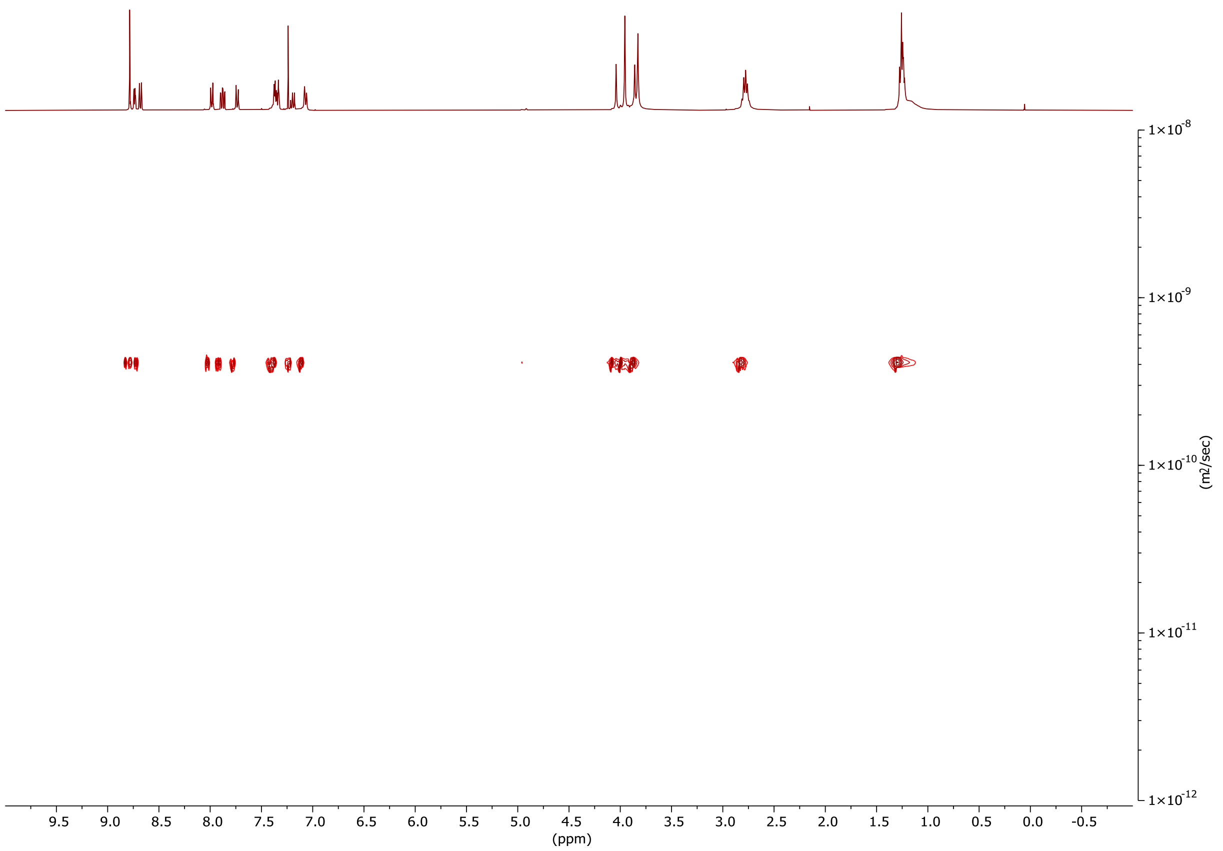

Figure S15. ^{1}H DOSY NMR spectrum of **9** in $CDCl_3$ at rt.

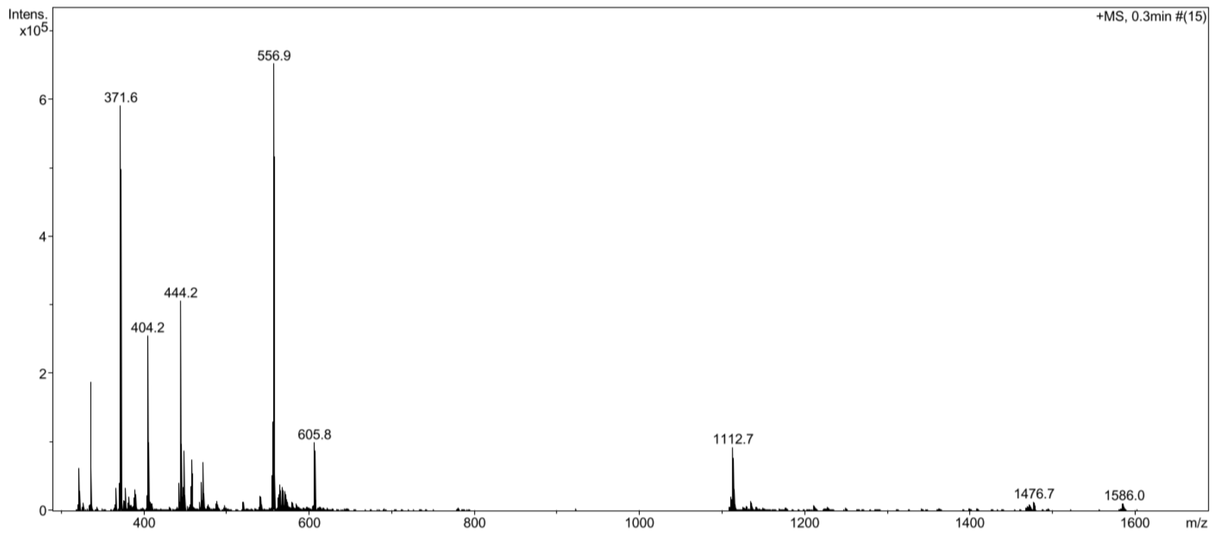

Figure S16. ESI MS spectrum of **9**.

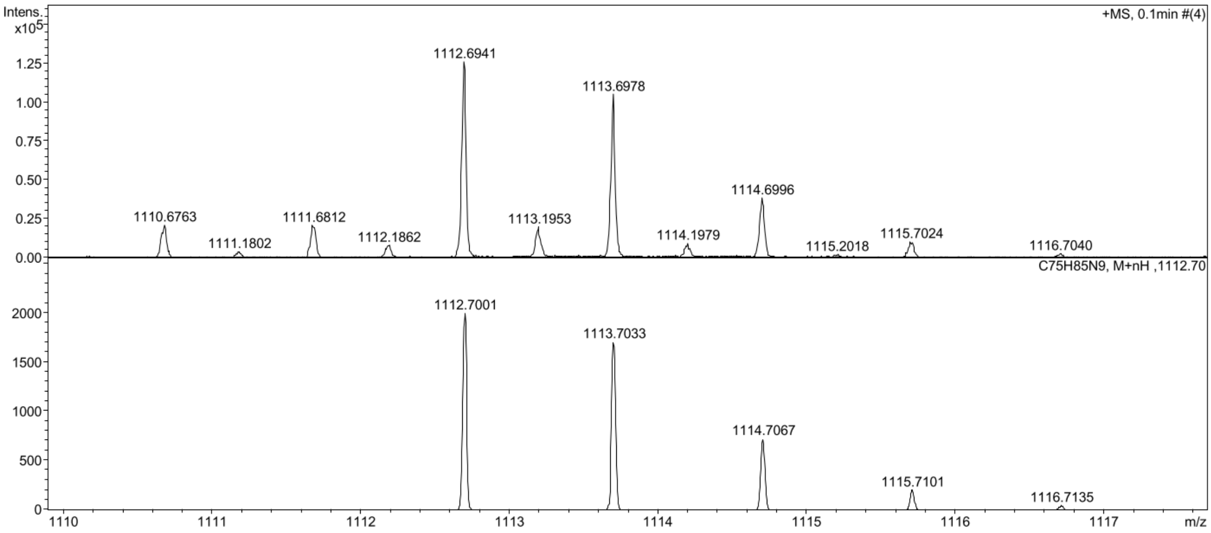

Figure S17. Measured and calculated ESI MS of **9** for $[M+H]^+$.

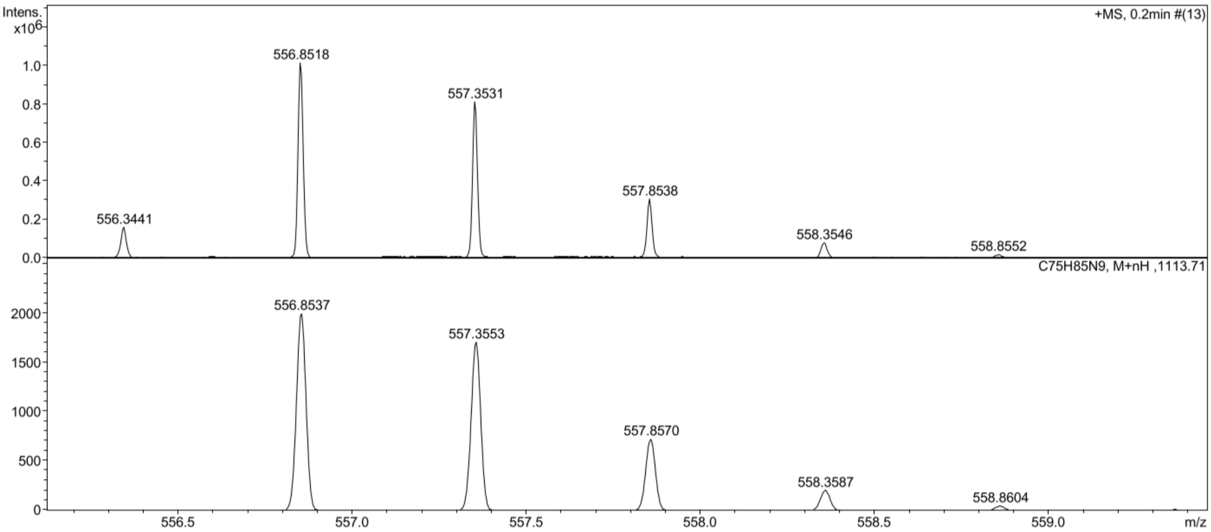

Figure S18. Measured and calculated ESI MS of **9** for $[M+2H]^{2+}$.

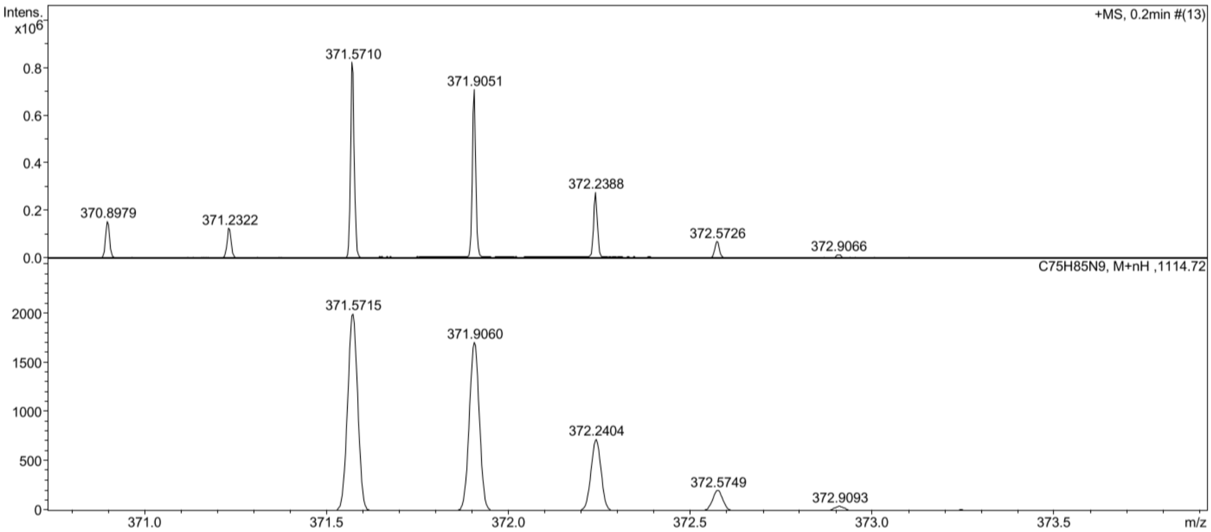

Figure S19. Measured and calculated ESI MS of **9** for $[M+3H]^{3+}$.

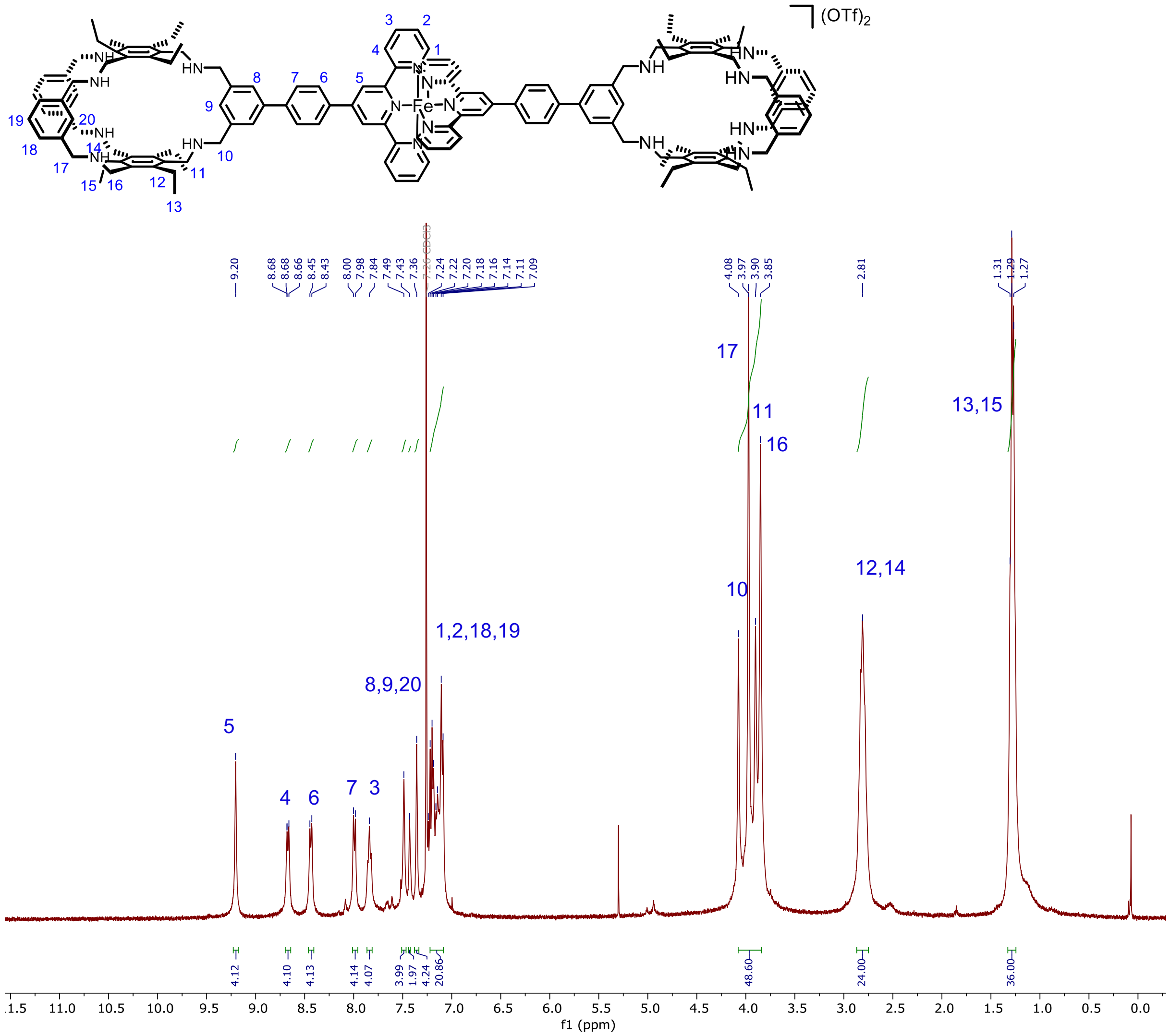

Figure S20. ^{1}H NMR spectrum of **10(OTf)$_2$** in $CDCl_3$ at rt.

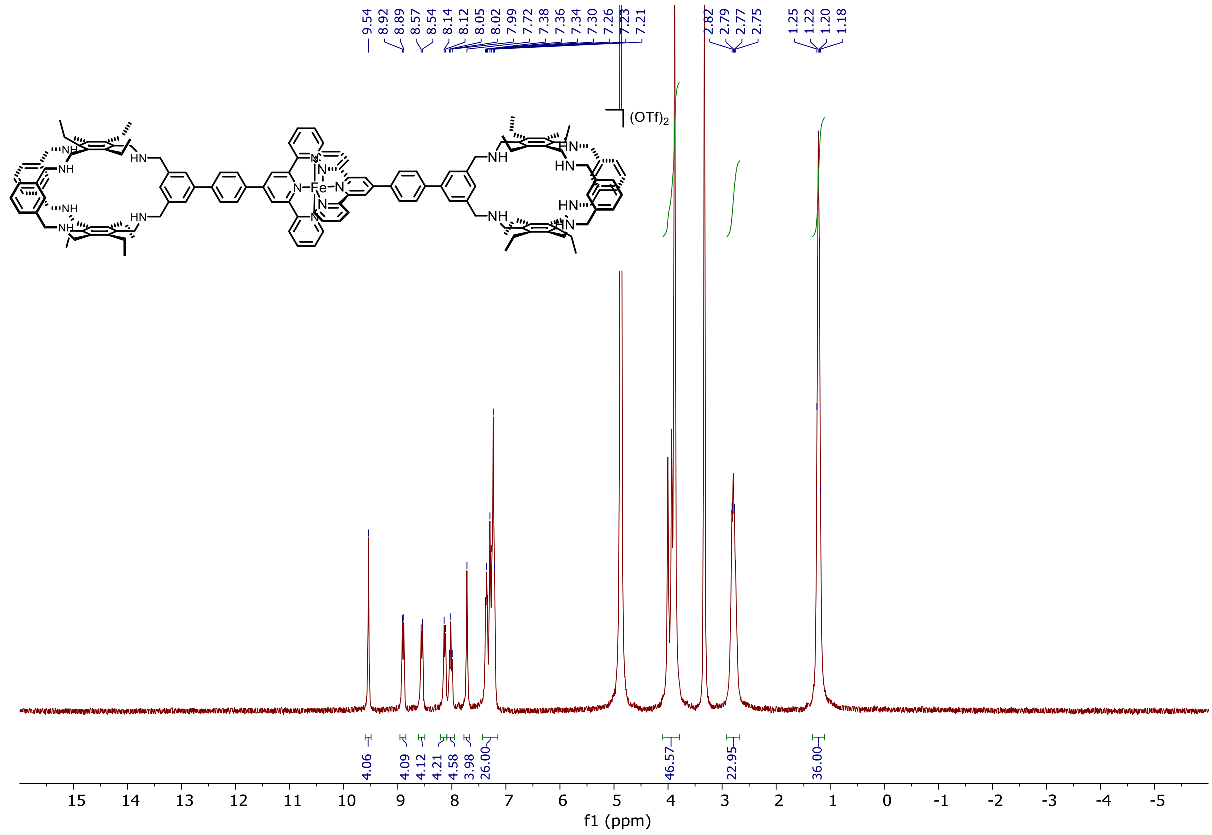

Figure S21. ^{1}H NMR spectrum of **10(OTf)$_2$** in CD_3OD at rt.

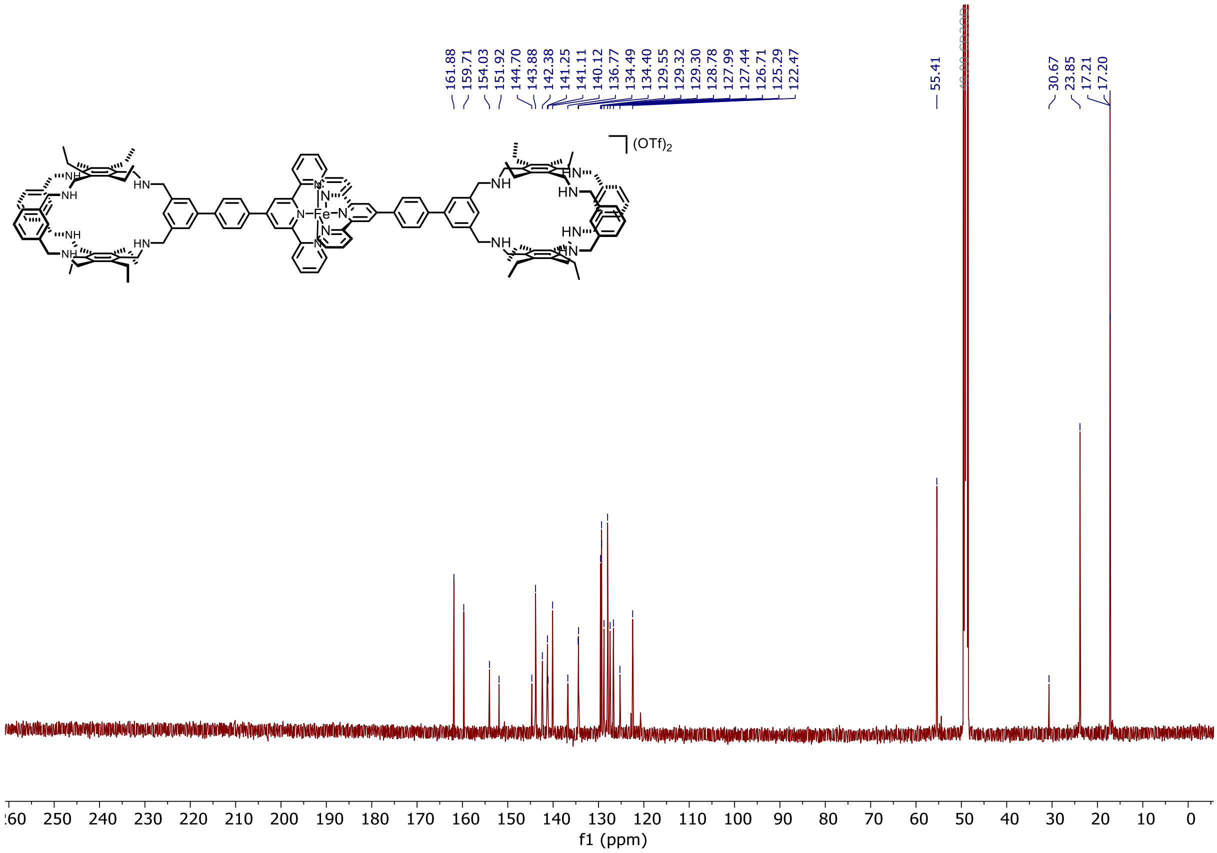

Figure S22. ^{13}C NMR spectrum of **10(OTf)$_2$** in CD_3OD at rt.

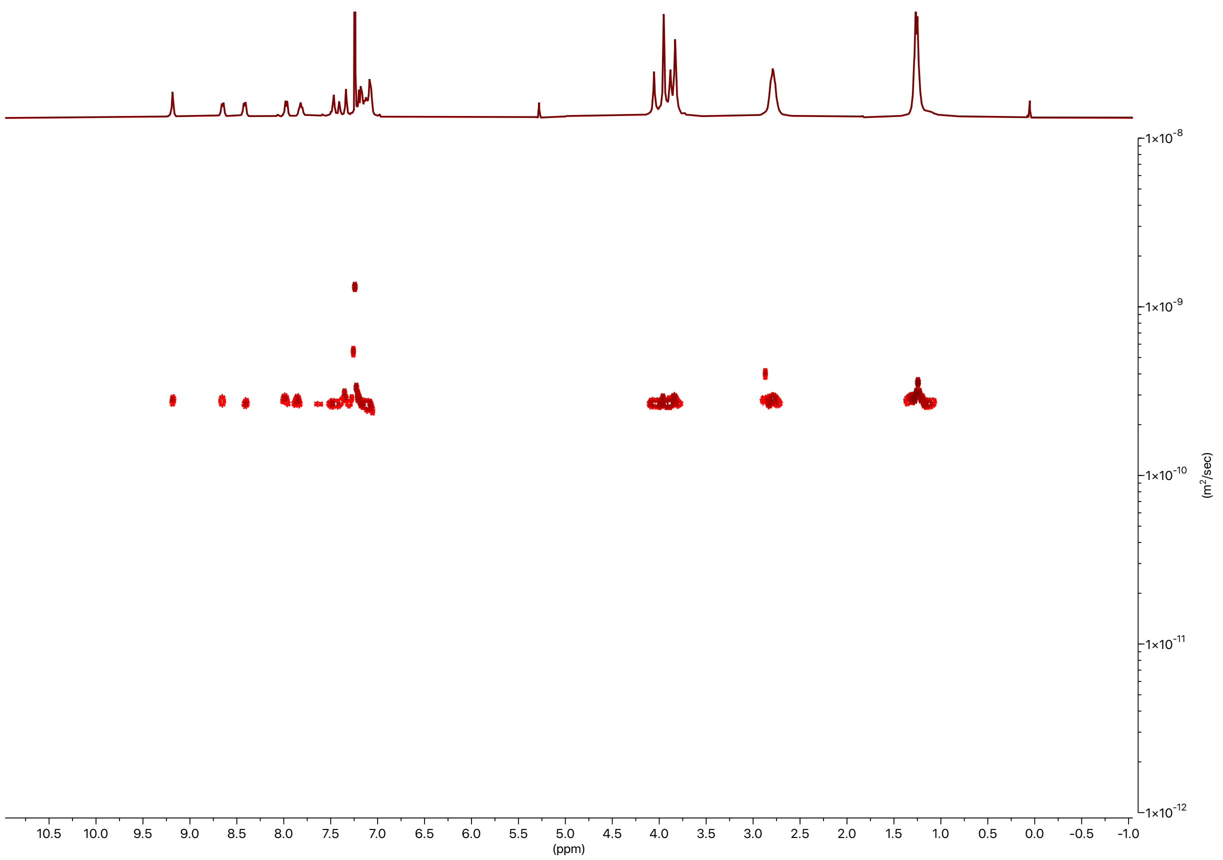

Figure S23. ^{1}H DOSY NMR spectrum of **10(OTf)₂** in $CDCl_3$ at rt.

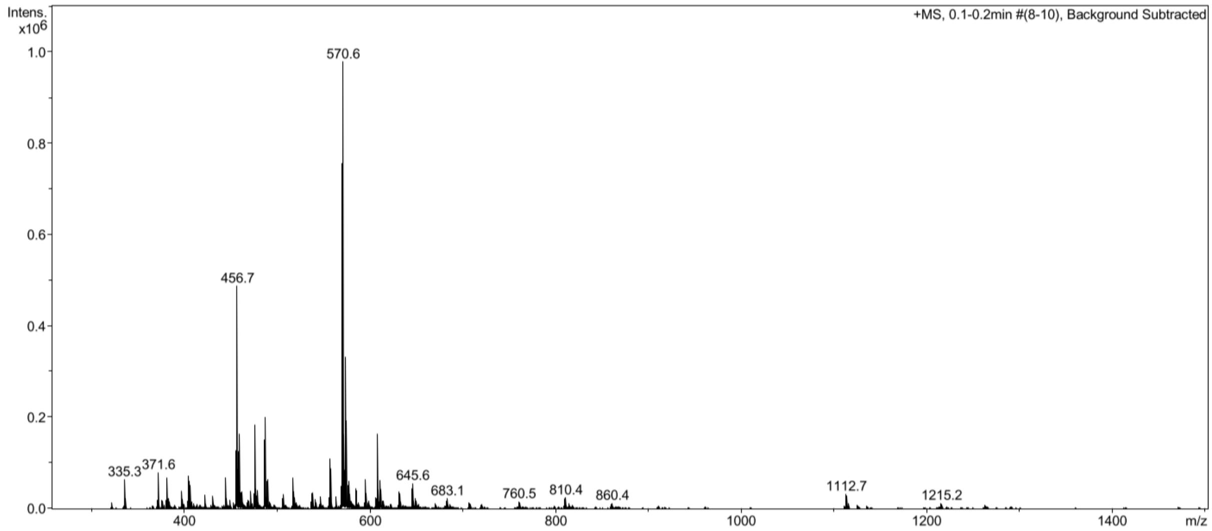

Figure S24. ESI MS spectrum of **10(OTf)₂**.

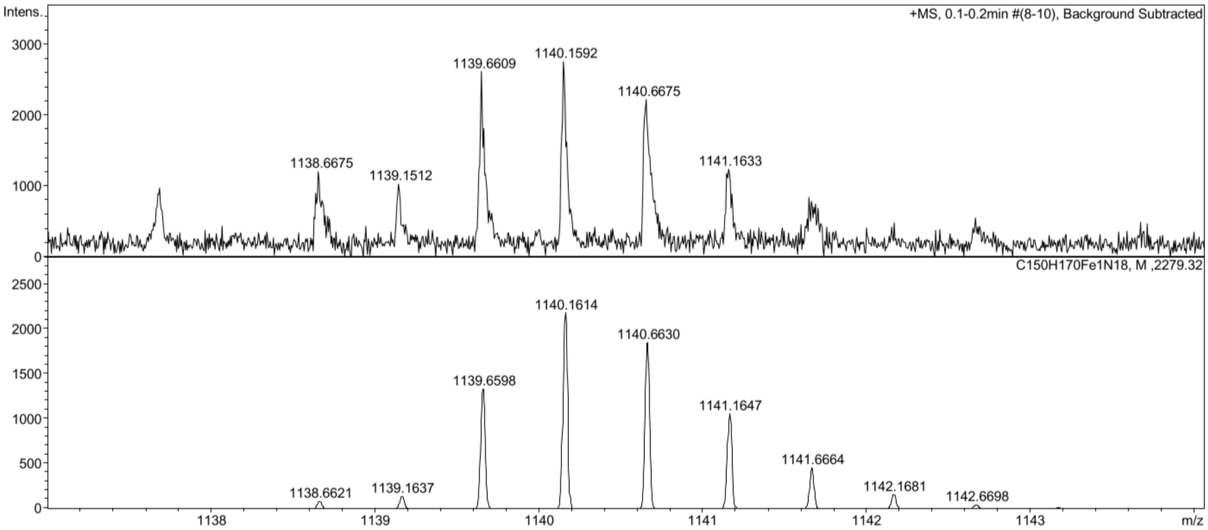

Figure S25. Measured and calculated ESI MS of **10(OTf)₂** for $[M]^{2+}$.

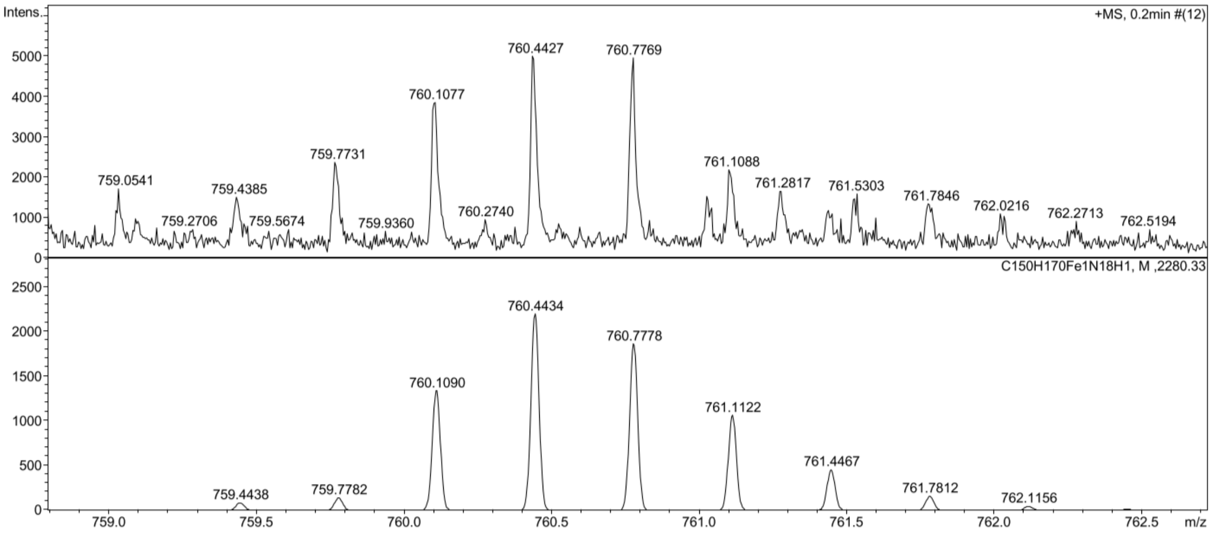

Figure S26. Measured and calculated ESI MS of **10(OTf)₂** for $[M+H]^{3+}$.

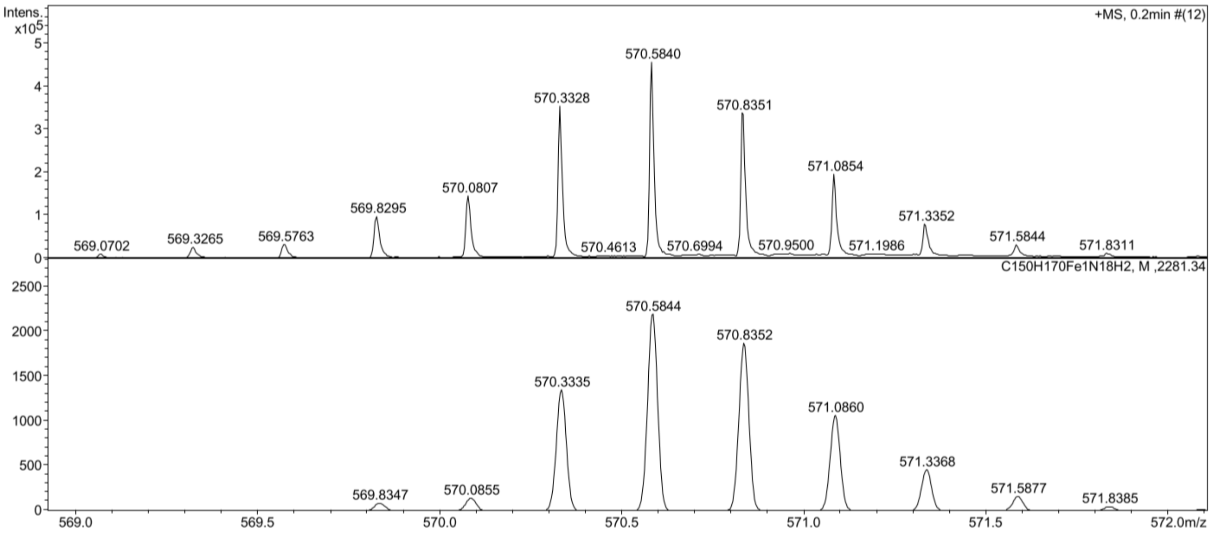

Figure S27. Measured and calculated ESI MS of **10(OTf)₂** for $[M+2H]^{4+}$.

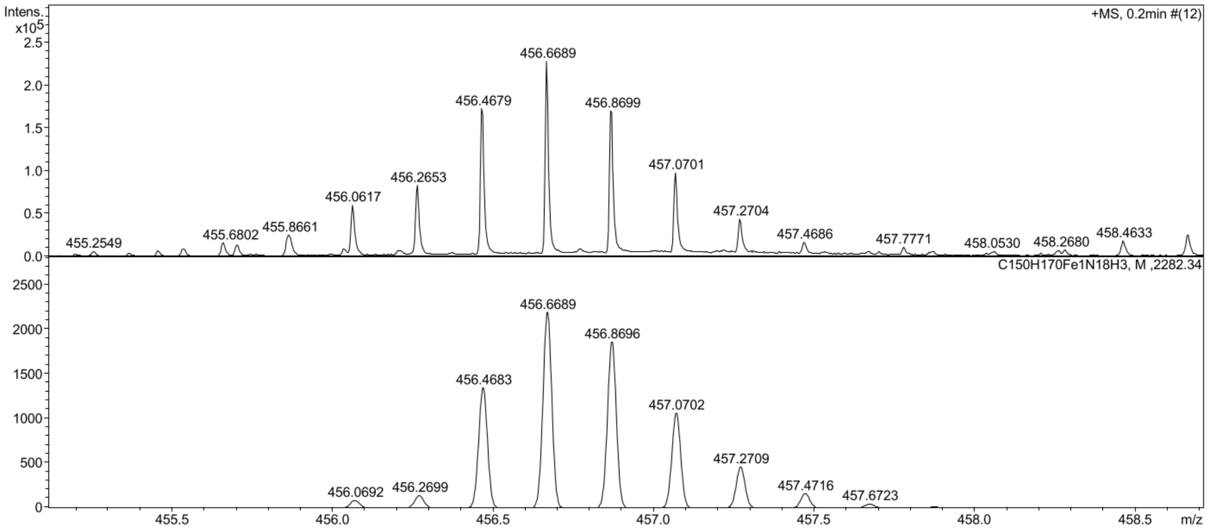

Figure S28. Measured and calculated ESI MS of **10(OTf)₂** for $[M+3H]^{5+}$.

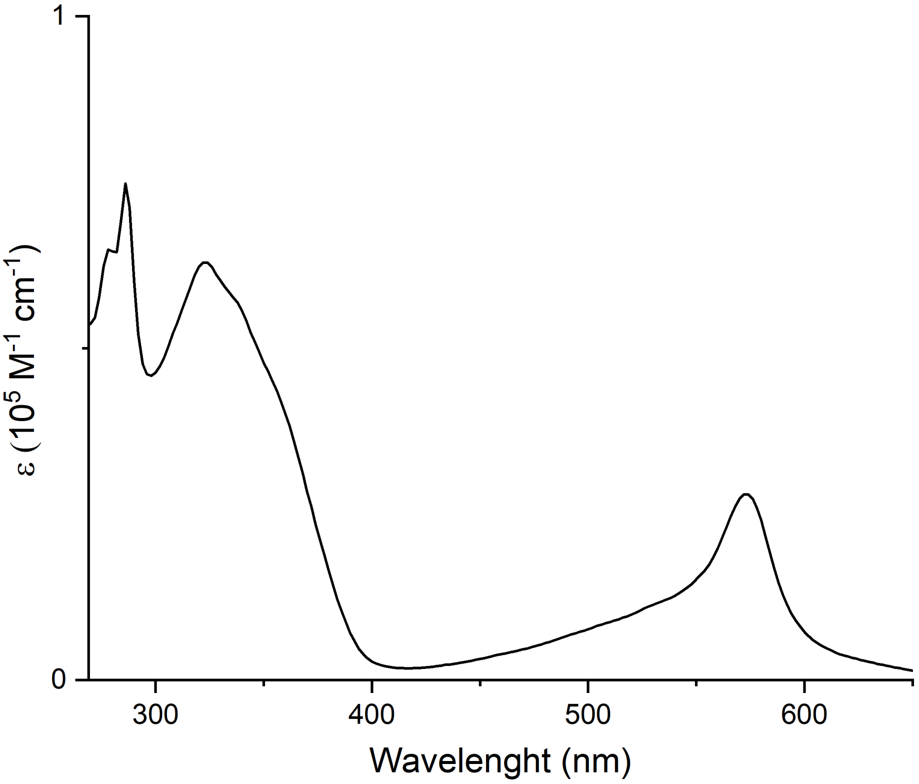

Figure S29. UV/vis Spectrum of **10(OTf)₂** recorded in dichloromethane.

4 Computational Details

The reported results build upon a series of electronic structure calculations on multiple levels of theory. The first set of calculations utilizes the CREST algorithm of Grimme and coworkers together with the semi-empirical GFN2-xTB method of the same group to explore the low-energy conformational space of isolated units of **9**.[3,4] In the description of the conducted procedures below this set of calculations is denoted CREST/xTB.

On top of the aforementioned conformational search, lowest energy structures of isolated units of **9** and $[Fe(terpy\text{-}Ph)_2]^{2+}$ as well as complete $\mathbf{10}^{2+}$ were refined by DFT geometry optimizations that were conducted with the ORCA program package in its version 4.2.[5] During all reported DFT calculations the generation of Coulomb integrals was accelerated by the Resolution-of-identity approximation with the def2/J basis set.[6-8] Dispersion effects were accounted for by the D3 approximation using Becke-Johnson damping (D3BJ)[9] while solvation effects were modeled by a conductor-like polarizable continuum model (C-PCM) with a dielectric constant of ε = 9:08 for CH_2Cl_2.[10] Finally, our reported DFT employed various combinations of the BP86 and the TPSS functionals with the def2-SVP basis and the def2-TZVP(-f) basis sets. In the description of the conducted procedures below these sets of calculations are denoted "Basis/Basis Set".[11-14] In cases, where no C-PCM description of solvation effects was enabled, "(no C-PCM)" will be added.

In the following the procedure for the generation of structures of $\mathbf{10}^{2+}$ without solvent molecules is described:

• Optimization of $[Fe(terpy\text{-}Ph)_2]^{2+}$ and **9** with xTB

• Optimization of $[Fe(terpy\text{-}Ph)_2]^{2+}$ with TPSS/def2-TZVP(-f)

• Optimization of **9** with BP86/def2-TZVP(-f)

• Connection of **9** and $[Fe(terpy\text{-}Ph)_2]^{2+}$ to yield starting structure of $\mathbf{10}^{2+}$

• Optimization of $\mathbf{10}^{2+}$ with TPSS/def2-SVP invoking only loose SCF and optimization convergence threshholds

• Optimization of $\mathbf{10}^{2+}$ with TPSS/def2-SVP with default SCF convergence thresholds but "LooseOPT" convergence thresholds for the geometry optimization.

In addition to the optimization of isolated $\mathbf{10}^{2+}$ in an implicit solvation model we optimized **9** in the presence of 4 explicit solvent molecules with CREST/xTB. However, during the optimization procedure, all 4 solvent molecules consistently left the cage to enable the π - π interaction described in the main manuscript.

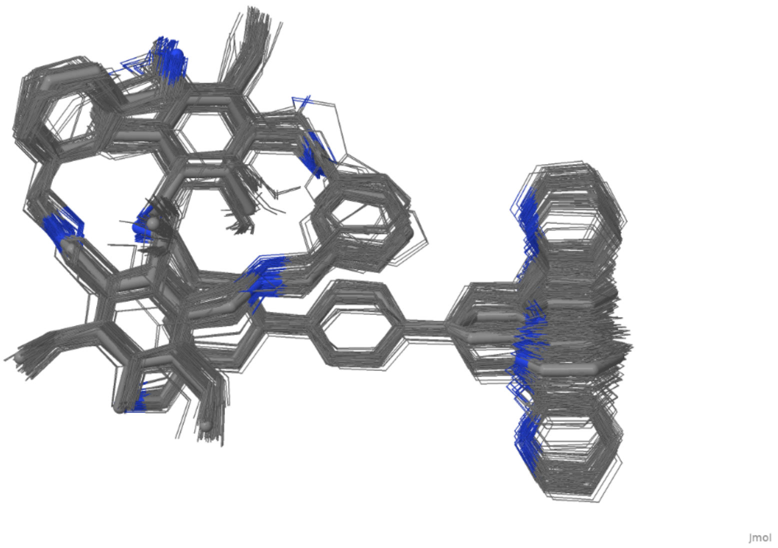

Figure S30. Overlay of 230 conformer structures of **9** that lie within an energy range of 5 kcal mol^{-1} according to CREST.

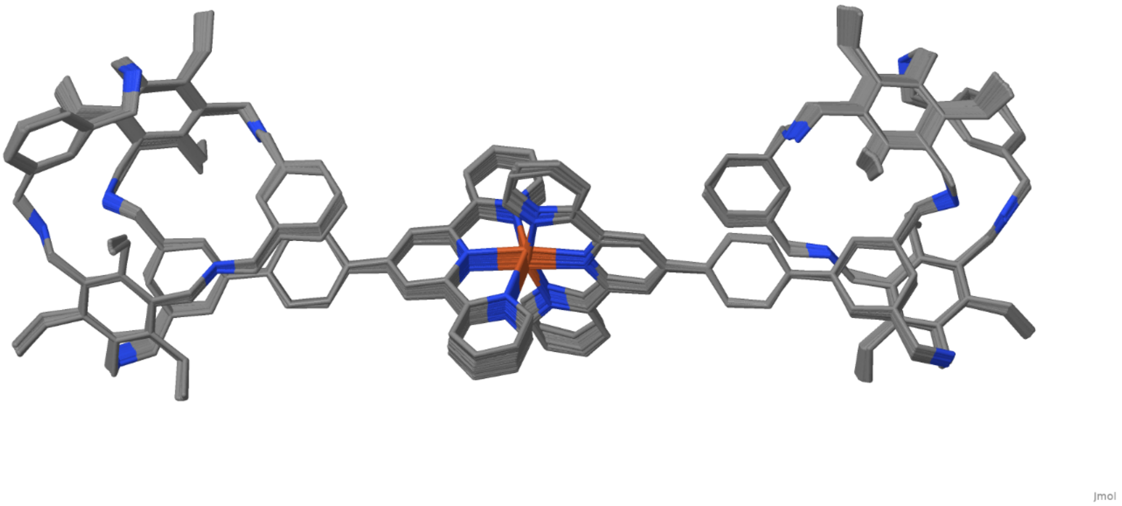

Figure S31. Overlay of the structure obtained from fusing optimized structures of **9** and $[Fe(terpy\text{-}Ph)_2]^{2+}$ (details see text) and the optimized structure of **10^{2+}** alongside the trajectory. The obvious similarity between the structures indicates only weak structural relaxation upon the connection of the two cage units.

5 References

[1] O. Francesconi, M. Gentili, C. Nativi, A. Ardá, F. J. Cañada, J. Jiménez-Barbero and S. Roelens, *Chem. Eur. J.*, 2014, **20**, 6081.

[2] B. Mondal, A. K. Ghosh and P. S. Mukherjee, *J. Org. Chem.*, 2017, **82**, 7783.

[3] P. Pracht, F. Bohle and S. Grimme, *Phys. Chem. Chem. Phys.*, 2020, **22**, 7169.

[4] C. Bannwarth, S. Ehlert and S. Grimme, *J. Chem. Theory Comput.*, 2019, **15**, 1652.

[5] F. Neese, WIREs Comp. *Mol. Sci.*, 2018, **8**, e1327.

[6] B. I. Dunlap, J. W. D. Connolly and J. R. Sabin, *J. Chem. Phys.*, 1979, **71**, 3396.

[7] O. Vahtras, J. Alml□of and M. W. Feyereisen, *Chem. Phys. Lett.*, 1993, **213**, 514.

[8] F. Weigend, *Phys. Chem. Chem. Phys.*, 2006, **8**, 1057.

[9] S. Grimme, J. Antony, S. Ehrlich and H. Krieg, *J. Chem. Phys.*, 2010, **132**, 154104.

[10] M. Cossi, V. Barone, R. Cammi and J. Tomasi, *Chem. Phys. Lett.*, 1996, **255**, 327.

[11] J. P. Perdew, *Phys. Rev. B*, 1986, **33**, 8822.

[12] A. D. Becke, *Phys. Rev. A*, 1988, **38**, 3098.

[13] J. Tao, J. P. Perdew, V. N. Staroverov and G. E. Scuseria, *Phys. Rev. Lett.*, 2003, **91**, 146401.

[14] F. Weigend and R. Ahlrichs, *Phys. Chem. Chem. Phys.*, 2005, **7**, 3297.
